# Layer‐Specific Astrocyte Morphological Responses in the CA3 Hippocampus Region During Piry Virus‐Induced Encephalitis

**DOI:** 10.1002/hipo.70085

**Published:** 2026-02-22

**Authors:** Diego de Almeida Miranda, Aline Andrade de Sousa, Renata Rodrigues dos Reis, Zaire Alves dos Santos, José Antonio Picanço Diniz, Pedro Fernando da Costa Vasconcelos, Cristovam Guerreiro Diniz, Daniel Clive Anthony, Dora Brites, Cristovam Wanderley Picanço Diniz, Daniel Guerreiro Diniz

**Affiliations:** ^1^ Laboratório de Investigações em Neurodegeneração e Infecção Universidade Federal do Pará, Instituto de Ciências Biológicas, Hospital Universitário João de Barros Barreto Belém Pará Brazil; ^2^ Laboratório de Biologia Molecular e Neuroecologia Instituto Federal de Educação Ciência e Tecnologia do Pará, Campus Bragança Bragança Pará Brazil; ^3^ Laboratório de Microscopia Eletrônica Instituto Evandro Chagas, Seção de Hepatologia Belém Pará Brazil; ^4^ Centro de Ciências Biológicas e da Saúde Universidade do Estado do Pará Belém Pará Brazil; ^5^ Department of Pharmacology, Laboratory of Experimental Neuropathology University of Oxford Oxford England UK; ^6^ Faculty of Pharmacy Universidade de Lisboa, Research Institute for Medicines (iMed.ULisboa) Lisbon Portugal; ^7^ Department of Biochemistry and Human Biology, Faculty of Pharmacy Universidade de Lisboa Lisbon Portugal; ^8^ Núcleo de Pesquisas em Oncologia Universidade Federal do Pará, Hospital Universitário João de Barros Barreto Belém Pará Brazil

**Keywords:** CA3 hippocampal region, encephalitis, hierarchical cluster and discriminant analysis, hippocampus, layer‐dependent astrocyte morphological response, Piry virus

## Abstract

Astrocytes from distinct hippocampal layers exhibit region‐specific morphological traits, which may be influenced by their local microenvironment. During viral encephalitis, these cells undergo dynamic changes that can reflect layer‐specific vulnerability. In this study, we characterized whether astrocytes from different CA3 hippocampal layers display distinct morphological responses to Piry virus‐induced encephalitis. Adult female Swiss mice were intranasally inoculated with the Piry virus and sacrificed at 20‐ or 40‐days post‐infection (dpi). GFAP+ astrocytes from the *Stratum lacunosum‐moleculare* (SLM) and *Stratum oriens* (SO) were three‐dimensionally reconstructed. Morphometric data were evaluated using hierarchical clustering, linear discriminant analysis (LDA), and generalized linear models. Immunohistochemistry confirmed widespread viral neuroinvasion across olfactory and limbic regions. Hierarchical clustering identified 3–4 morphotypes per layer and time point with robust internal consistency, and LDA validated cluster assignments with high accuracy (> 91%). At 20 dpi, SLM astrocytes displayed significantly greater morphological complexity than SO astrocytes, whereas at 40 dpi responses were more heterogeneous, indicating temporal diversification of astrocytic reactivity. These findings provide an observational description of layer‐ and time‐dependent astrocyte morphological plasticity during viral encephalitis. They underscore the value of morphometric and multivariate analyses for dissecting glial heterogeneity, while highlighting the need for future studies to determine the functional significance of these morphotypes.

**Abbreviations**
CA3: *Cornu Ammonis 3*
CEPAE‐UFPA: Animal Research Ethics Committee
CNS: central nervous system
df: degrees of freedom
dpi: day post‐infection
GFAP: glial fibrillary acidic protein
Hp: hippocampus
IEC‐Pará: Evandro Chagas Institute
k‐Dim: fractal dimension
LDA: Linear discriminant analysis
MANOVA: Multivariate Analysis of Variance
MMI: multimodality index
MolDG: molecular layer of the dentate gyrus
SLM: *Stratum lacunosum‐moleculare*
SO: *Stratum oriens*
Va: vertex type a
Vb: vertex type b
Vc: vertex type c

## 1 Introduction

Viral encephalitis is a severe inflammatory condition of the central nervous system (CNS) associated with significant morbidity and mortality (Bohmwald et al. 2021; Pavlou et al. 2024; Venkatesan et al. 2019). While neurons are primary targets, increasing evidence highlights glial cells, particularly astrocytes, as central mediators of disease outcomes (Kaur et al. 2023; Li et al. 2021; Mielcarska et al. 2021; Mora et al. 2024; Steardo and Scuderi 2023). Astrocytes support CNS homeostasis and undergo reactive astrogliosis during infection, with changes in morphology, gene expression, and function that can either mitigate or exacerbate neuropathology (Hosseini and Korte 2023; Soung and Klein 2018).

Astrocyte responses are not uniform across the brain. Regional and laminar microenvironments, including synaptic architecture and neurotransmitter tone, appear to influence astrocytic reactivity (Karpf et al. 2022; Lanjakornsiripan et al. 2018). In the hippocampus, the *Cornu ammonis 3* (CA3) *Stratum lacunosum‐moleculare* (SLM) receives glutamatergic input from the entorhinal cortex, whereas the *Stratum oriens* (SO) is enriched in GABAergic interneurons (Freund and Antal 1988; Megías et al. 2001; Tzilivaki et al. 2023). These contrasting inputs provide a framework to investigate whether local context is associated with distinct astrocytic responses to injury or infection. However, it is important to note that such associations remain correlative in nature and require additional approaches (e.g., synaptic marker colocalization or neurotransmitter release assays) to establish causal links.

The Piry virus, a Rhabdoviridae arbovirus with consistent tropism for the limbic system, provides a suitable model to explore astrocyte responses in viral encephalitis (da Silva Creão et al. 2021; de Sousa et al. 2015). Previous work demonstrated robust astrocytic and microglial changes in this model. Here, we aimed to describe whether astrocytes in excitatory (SLM) and inhibitory (SO) CA3 domains exhibit distinct morphological trajectories following Piry virus infection, using 3D morphometric reconstruction and multivariate analyses at 20‐ and 40‐days post‐infection. Rather than testing a mechanistic hypothesis, this study provides an observational characterization of layer‐specific astrocytic morphological adaptations during encephalitis progression.

## 2 Methods

**FIGURE 1:**
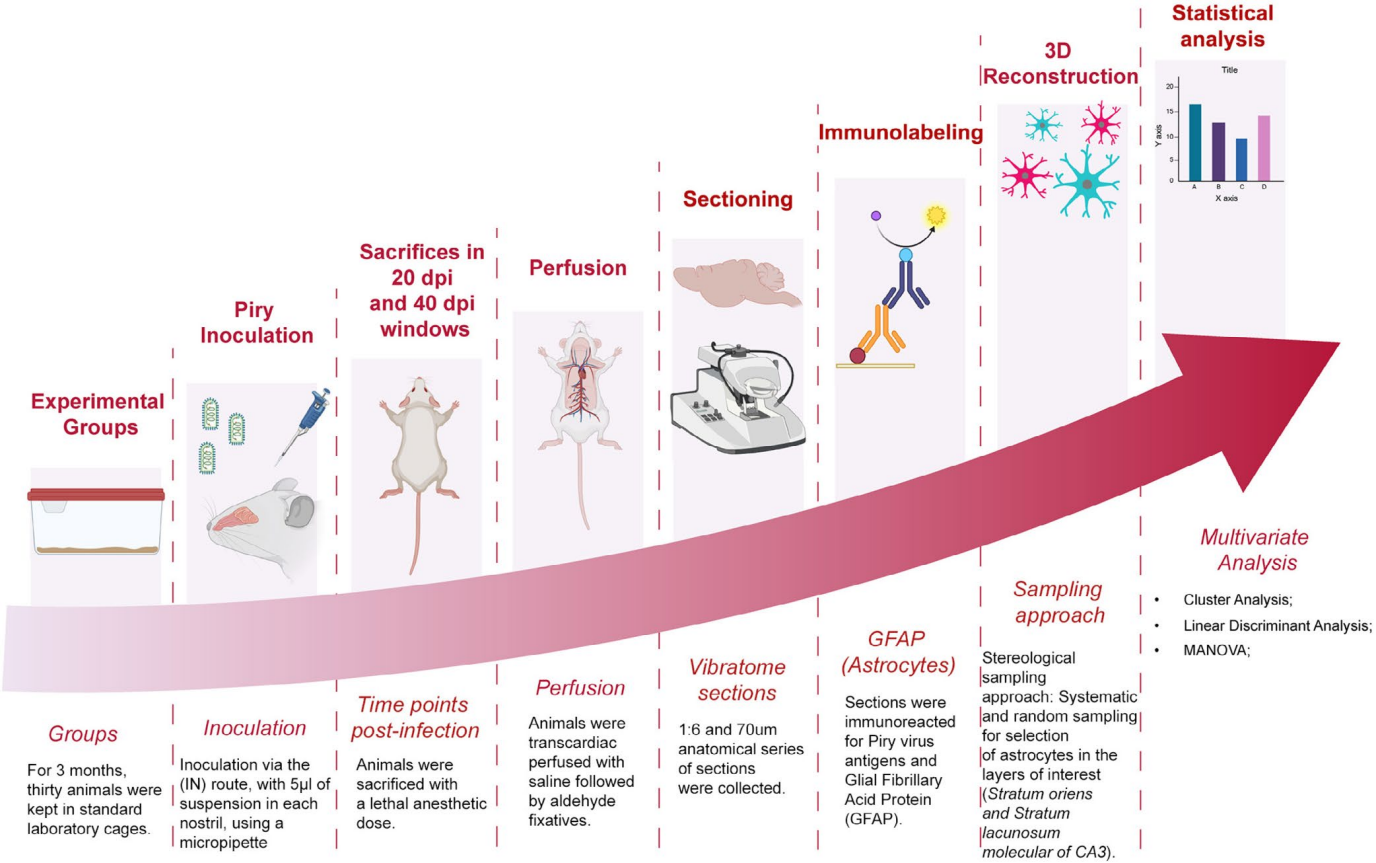
Schematic overview of the experimental workflow used to investigate astrocyte responses to Piry virus infection in the CA3 region of the hippocampus. Thirty adult Swiss albino mice were housed in standard laboratory cages for three months before intranasal inoculation with the Piry virus (5 μL per nostril). Animals were sacrificed at two post‐infection time points (20 and 40 days) using a lethal anesthetic dose. Transcardiac perfusion was performed with heparinized saline, followed by aldehyde‐based fixatives. Vibratome sectioning yielded 70‐μm‐thick coronal slices in the 1:6 anatomical series. Sections were immunolabeled for Piry virus antigens and the astrocytic marker glial fibrillary acidic protein (GFAP). Astrocytes were selected using a stereological sampling approach that involved systematic and random sampling from the *Stratum lacunosum‐moleculare* and *Stratum oriens* of the CA3 hippocampal subfield. Three‐dimensional reconstructions of astrocytes were generated and analyzed using multivariate statistical techniques, including cluster analysis, chi‐square tests, linear discriminant analysis, and MANOVA.

This study tested the hypothesis that astrocyte morphological responses to Piry virus infection in the CA3 region of the hippocampus show layer specificity. To this end, astrocytes immunolabeled for glial fibrillary acidic protein (GFAP) in the SLM and SO of CA3 were reconstructed in brain sections from mice inoculated with Piry virus and compared to those from the same layers with non‐infected homogenate. Figure 1 illustrates the experimental timeline in graphical form. The methods used in the original study to generate the slide collection are described below.

### 2.1 Animals, Experimental Design, and Virus Inoculation

Thirty adult female Swiss albino mice, 2 months old, were obtained from the Evandro Chagas Institute (IEC—Pará) and handled in accordance with the NIH's Principles of Laboratory Animal Care.

All experimental procedures were conducted in accordance with institutional and national guidelines for the care and use of laboratory animals and were approved by the Ethics Committee on Animal Research of the Federal University of Pará (protocol no. [222‐14]). The study used adult female BALB/c mice, 2 months old at the time of infection, housed in groups of five per cage under a 12:12 h light–dark cycle with controlled temperature (22°C ± 2°C) and ad libitum access to food and water. Female mice were selected to minimize stress‐induced variability commonly observed in male colonies due to territorial aggression and hierarchical disputes. A minimum of five animals was included in each experimental group (control and infected) for each survival time point (20‐ and 40‐days post‐infection), ensuring adequate biological replication for statistical analyses. The mice were housed in standard polypropylene cages (32 × 39 × 16.5 cm) lined with wood shavings and provided with unrestricted access to food and water. Each cage housed approximately 15 animals, and aside from social interaction, no additional cognitive or physical stimulation was provided. After 3 months in this environment, animals were infected (intranasally inoculated) with the Piry arbovirus and sacrificed at 20 or 40 days post‐infection (dpi). Figure 1 illustrates the experimental timeline in graphical form.

Piry virus inoculation was performed at the Evandro Chagas Institute under institutional biosafety protocols inside Class II B2 laminar flow hoods, as previously described (de Sousa et al. 2015). Briefly, newborn Swiss albino mice were intracerebrally injected with 10 μL of a viral suspension containing the Piry virus and killed after exhibiting symptoms of encephalitic infection. The infected brains were homogenized (0.2 g in 0.8 mL) of sterile phosphate‐buffered saline supplemented with penicillin (100 U/mL) and streptomycin (100 μg/mL) to prevent bacterial contamination. The suspension was centrifuged at 10,000 × g for 15 min at 4°C, and the resulting supernatant was used as a viral solution. The inoculum titration was standardized at a dilution of 10^−5^ (v/v), considered effective for inducing subacute encephalitis with sufficient survival for brain morphological analyses at different times post‐infection. Inoculation was performed intranasally, with 5 μL of the viral suspension administered to each nostril (totaling 10 μL per animal) using manual micropipettes. This method mimics the natural route of infection, allowing for progressive neuroinvasion through the olfactory bulb and into limbic areas, such as the hippocampus. At 20 or 40 dpi, animals were anesthetized with intraperitoneal Avertin (tribromoethanol) and perfused transcardially with 0.9% heparinized saline for 10 min, followed by 4% paraformaldehyde for 40 min. Brains were extracted, sectioned at 70 μm thickness using a vibratome, and stored in 2% paraformaldehyde for immunohistochemical processing.

### 2.2 Immunohistochemistry

To evaluate the effects of Piry virus infection at 20 and 40 dpi, immunohistochemistry was used to label Piry virus antigens and astrocytes. For viral antigen detection, a Piry virus‐specific antibody produced by the Arbovirus Department at IEC was used, following the protocol of Braga and Santos (Braga and Santos 2008). For 3D reconstructions, GFAP immunolabeling was performed as described previously (de Sousa et al. 2015). GFAP is a key structural protein in astrocytes and serves as a reliable marker of astrocyte reactivity, with elevated expression commonly observed in inflammation, brain injury, and neurodegenerative conditions (Boulton and Al‐Rubaie 2025; Nourbakhsh et al. 2021).

### 2.3 Anatomical Landmarks and Morphometry

**FIGURE 2:**
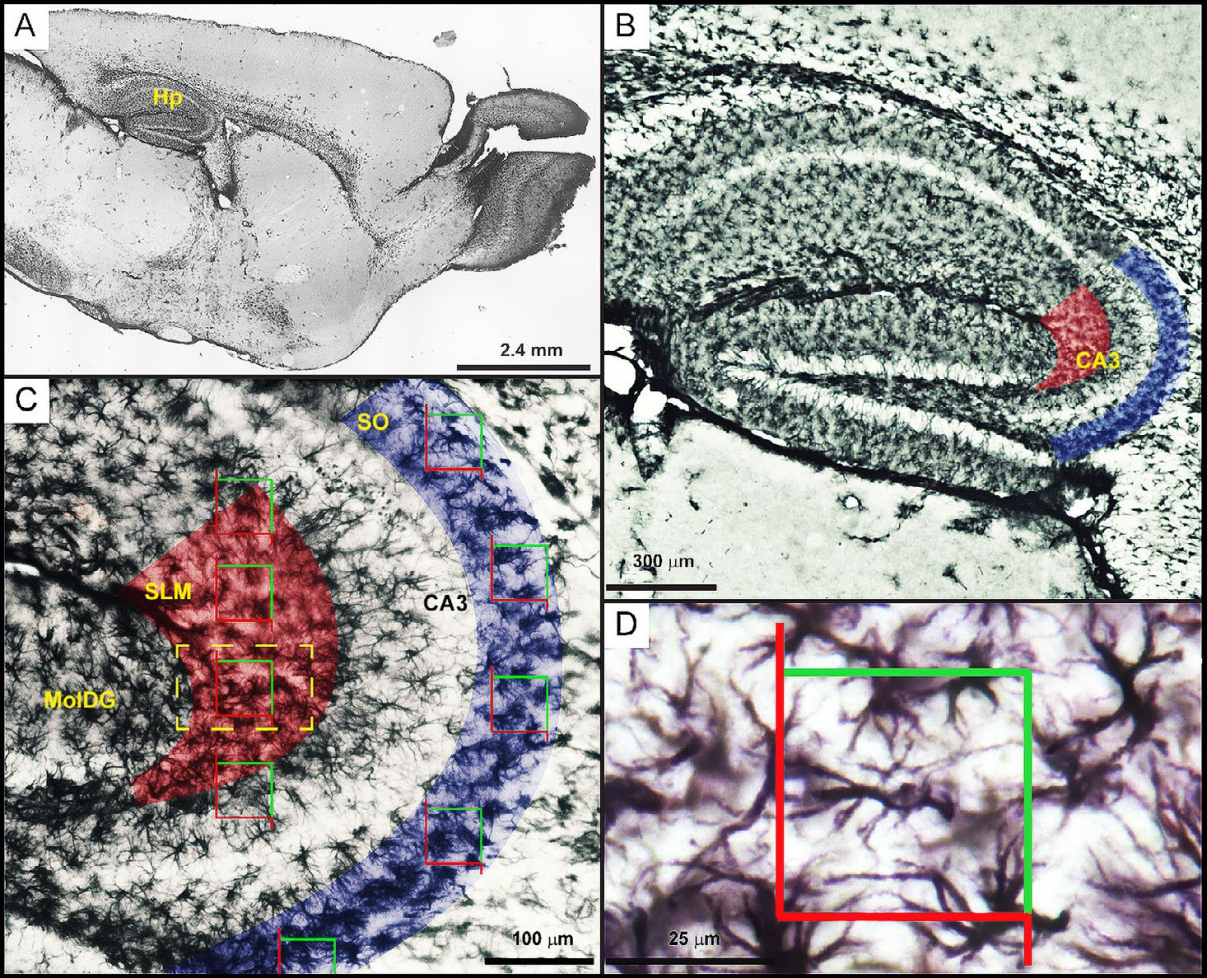
Photomicrographs of the hippocampal formation of an adult Swiss Albino mouse, illustrating the morphological criteria used to delineate the CA3 region of the hippocampus (Hp). The hippocampal sublayers corresponding to the *Stratum lacunosum‐moleculare* (SLM) and *Stratum oriens* (SO) are highlighted in red and blue, respectively. Sampling boxes outlined in red and green were generated using the Stereoinvestigator software and its Optical Fractionator module to ensure that all regions of interest had an equal probability of contributing to the sample. Accordingly, the number of boxes was proportional to the dimensions of the target area from which astrocytes were selected for 3D reconstruction. The total number of boxes was determined based on pre‐calculated individual and total contour areas, the target number of cells to be reconstructed, and a proportional rule designed to yield 30 astrocytes per animal. (A) Low‐magnification image of the entire Hp formation. (B) Hp laminar organization, where the pyramidal layers of Ammon's horns (CA1, CA2, and CA3) and the granule cell layer of the dentate gyrus appear as lightly stained bands with low cellular density. The CA3 borders with the polymorphic layer of the dentate gyrus (green arrow), and CA2 (red arrow) are visible. (C) The intermediate magnification (10×) view of the CA3 region shows systematically and randomly distributed sampling boxes generated by the Optical Fractionator. (D) High‐magnification view (scale bar: 25 μm) of a GFAP^+^ astrocyte within a sampling box. MolDG, molecular layer of the dentate gyrus.

In GFAP‐immunolabeled sections, hippocampal CA3 sublayers were identified in parasagittal sections based on standard anatomical landmarks (see Figure 2). For anatomical delineation of CA3 sublayers for astrocyte reconstruction, all sections were counterstained with cresyl violet, which enabled precise delineation of the cytoarchitectural frontiers of the hippocampal layers based on neuronal soma density and neuropil texture. The SLM was defined as the superficial, lightly stained band adjacent to the hippocampal fissure, positioned above the *Stratum radiatum* and below the molecular layer of the dentate gyrus, characterized by sparse neuronal profiles and distal dendritic tufts of CA3 pyramidal neurons. The SO, in turn, was identified as the layer located immediately below the pyramidal cell layer, extending toward the alveus and containing scattered interneurons, basal dendrites, and commissural fibers. The upper boundary of the SO was marked by the dense pyramidal layer, and the deep boundary by the appearance of myelinated fibers of the alveus (Paxinos and Franklin 2001).

To ensure anatomical consistency, astrocytes were sampled from the same septotemporal (dorsoventral) level of the CA3 region, corresponding to Bregma −2.8 to −3.4 mm according to the mouse brain atlas (Paxinos and Franklin 2001). These explicit anatomical criteria now clarify the regional selection process and ensure reproducibility across samples.

### 2.4 Sampling Grid and Stereological Design

The grid used for quantification was established within the *StereoInvestigator* software (MBF Bioscience), which follows an unbiased, systematic, and random sampling stereological approach. This method ensures that each region of interest has an equal probability of being sampled, minimizing spatial bias in cell selection. The step length of the grid was defined based on pilot sampling to balance efficiency and representativeness across the CA3 field. The number of reconstructed astrocytes per section was proportional to the area of each sampled region, and at least 30 astrocytes per layer per animal were reconstructed to achieve a representative morphometric dataset.

### 2.5 Experimental Design and Sample Size

Each experimental group consisted of at least five animals per time point (control and infected, 20 and 40 dpi). Morphometric analyses were based on the average values per animal, ensuring that the statistical comparisons and effect size estimations reflected biological replication at the animal level rather than at the cell level. This approach meets the requirement of using mice as the experimental unit, consistent with the editor's recommendation. All significant differences and comparative effect sizes were therefore established using data from a minimum of five animals per experimental group. Astrocytes from the *Stratum lacunosum‐moleculare* (SLM) and *Stratum oriens* (SO) were reconstructed using systematic random sampling, with 30 cells analyzed per animal. Contours were defined using a 4× objective, and 3D reconstruction was performed using a 100× oil‐immersion lens (PLANFLUOR, NA 1.3; depth factor = 0.2 μm; Nikon, Japan). Only astrocytes with somata within the sampling box and well‐preserved branching were selected. If no suitable cell was found within the box, the nearest qualifying astrocyte was chosen.

Shrinkage in the Z‐axis was corrected using a 1.75× factor, as recommended by Carlo and Stevens (Carlo and Stevens 2011). Morphological reconstructions were performed by a blinded evaluator. Vascular astrocytes and cells with incomplete labeling or truncated processes were excluded. When astrocytes were absent from a given sampling box, the closest eligible astrocyte was reconstructed. Fifteen morphometric variables were extracted using *NeuroExplorer* software (see Table S1). These metrics informed subsequent hierarchical clustering and discriminant analysis.

### 2.6 Photomicrographs, Image Post‐Processing, and Representative Cells

Photomicrographs were taken using a Nikon Eclipse 80i microscope with a Microfire digital camera. Image brightness and contrast were adjusted in Adobe Photoshop and assembled in Illustrator. Representative astrocytes for each cluster were selected based on Euclidean distances in Z‐score space. The nearest‐neighbor approach was used to highlight morphological similarity. R with Multivariate Analysis (DOE 2023) and cluster packages (Maechler et al. 2023) helped to visualize typical and atypical astrocyte profiles within each group. A Euclidean distance matrix was constructed to select representative cells for each group using standardized data (Z‐scores), based on the multimodal variables that most significantly contributed to cluster formation. The analysis employed the shortest distance criterion to identify the cell that best represented each cluster. The cell with the shortest distance corresponds to the one exhibiting the lowest dissimilarity among the cells within the cluster, thus most accurately reflecting the shared morphological features of that group.

### 2.7 Statistical Analyses

**FIGURE 3:**
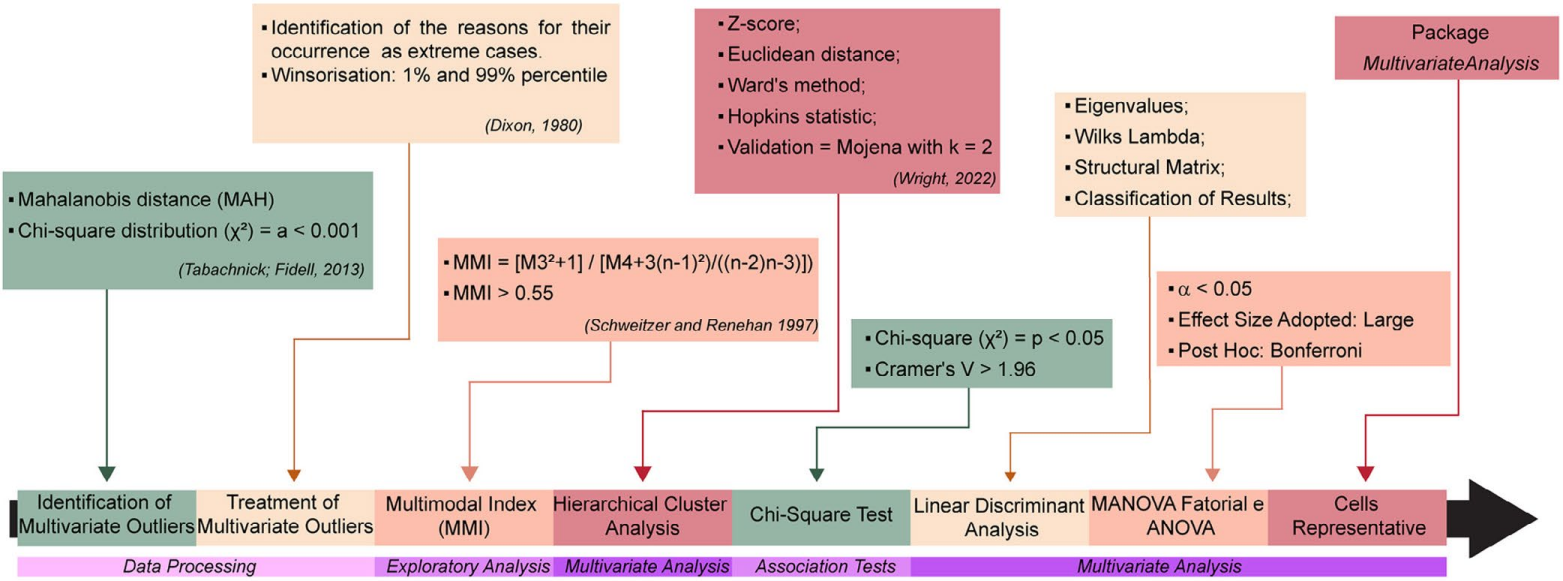
Statistical Workflow. Data processing and statistical analyses, from the identification and treatment of multivariate outliers (Mahalanobis distance and chi‐square distribution) to exploratory analyses (multimodality index), multivariate procedures (hierarchical cluster analysis, linear discriminant analysis, MANOVA, and ANOVA), and association tests (chi‐square and Cramer's V). The diagram also summarizes the statistical criteria applied, methodological references, software used for statistical processing and image editing, and the final step of identifying representative astrocytic cells.

Univariate, bivariate, and multivariate statistical analyses were conducted to examine relationships among morphometric parameters, experimental group, time point, and hippocampal layer. Multivariate outliers were detected using Mahalanobis distance (*p* < 0.001) and reviewed for potential data entry errors or biological divergence. Winsorisation (5th and 95th percentiles) was applied to reduce the influence of extreme values. Variables with a multimodality index (MMI) > 0.55 were retained. Hierarchical clustering was performed using Ward's method and Euclidean distances on Z‐score standardized variables. Cluster validity was assessed using Hopkins' statistics and cophenetic correlation coefficients (Figure 3). The optimal cut‐off point in the dendrogram was identified using Mojena's criterion (Mojena 1977), with a reference coefficient (*k* = 2) selected based on the distribution and stability of cluster solutions.

Figure 3 illustrates the complete workflow of statistical analyses, encompassing: Identification and treatment of multivariate outliers (Mahalanobis distance, *χ* ^2^ distribution, Winsorisation); Exploratory examination of variable distribution using MMI; Hierarchical clustering and validation (Ward's method, Euclidean distance, Hopkins' statistic, Mojena criterion); Association testing (*χ* ^2^, Cramer's V); Linear discriminant and multivariate analyses (*MANOVA*, *ANOVA*); Final identification of representative astrocytic morphotypes (See also Table S2). It provides a coherent, step‐by‐step representation that significantly enhances understanding for readers without extensive statistical background and therefore should be retained in the Methods section.

Linear discriminant analysis (LDA) validated cluster assignments and identified morphometric variables contributing to group separation. Scatter plots were generated using *SPSS* and *BioEstat*. Finally, a factorial *MANOVA* was performed in *JASP* (2024) with fixed factors including experimental group (control or infected), time point (20 or 40 dpi), and CA3 layer (SLM or SO). Assumptions of multivariate normality, homogeneity, and independence were checked, and results were interpreted cautiously given *MANOVA's* known robustness to minor violations (Finch and French 2015; Hair et al. 2014; Tabachnick and Fidell 2013).

## 3 Results

Morphometric analyses of astrocytes in the *Stratum lacunosum‐moleculare* (SLM) and *Stratum oriens* (SO) of the hippocampal CA3 region during viral encephalitis were performed using two complementary approaches. The first approach assessed the gross morphological features of control and post‐infection astrocyte populations without subgrouping by morphotype. The second approach classified astrocytes into distinct morphotypes via hierarchical clustering based on their morphometric profiles. These classifications were subsequently validated through linear discriminant analysis (LDA). A representative variable common to all clusters was then used to determine whether viral infection exerted differential effects on specific astrocyte morphotypes within each hippocampal layer. Section 3.1 presents the general (non‐clustered) analysis, while Sections 3.2 and onwards detail the morphotype‐based approach.

### 3.1 Gross Morphometric Analysis of GFAP‐Positive Astrocytes in the CA3 Hippocampus Following Viral Infection

Quantitative comparisons between control and infected animals at 20‐ and 40‐days post‐infection (dpi) revealed significant infection‐induced alterations in key astrocyte structural parameters. In both the SLM and SO, infected groups displayed increased total process length (Length SUM), number of segments, and convex hull measures.

**FIGURE 4:**
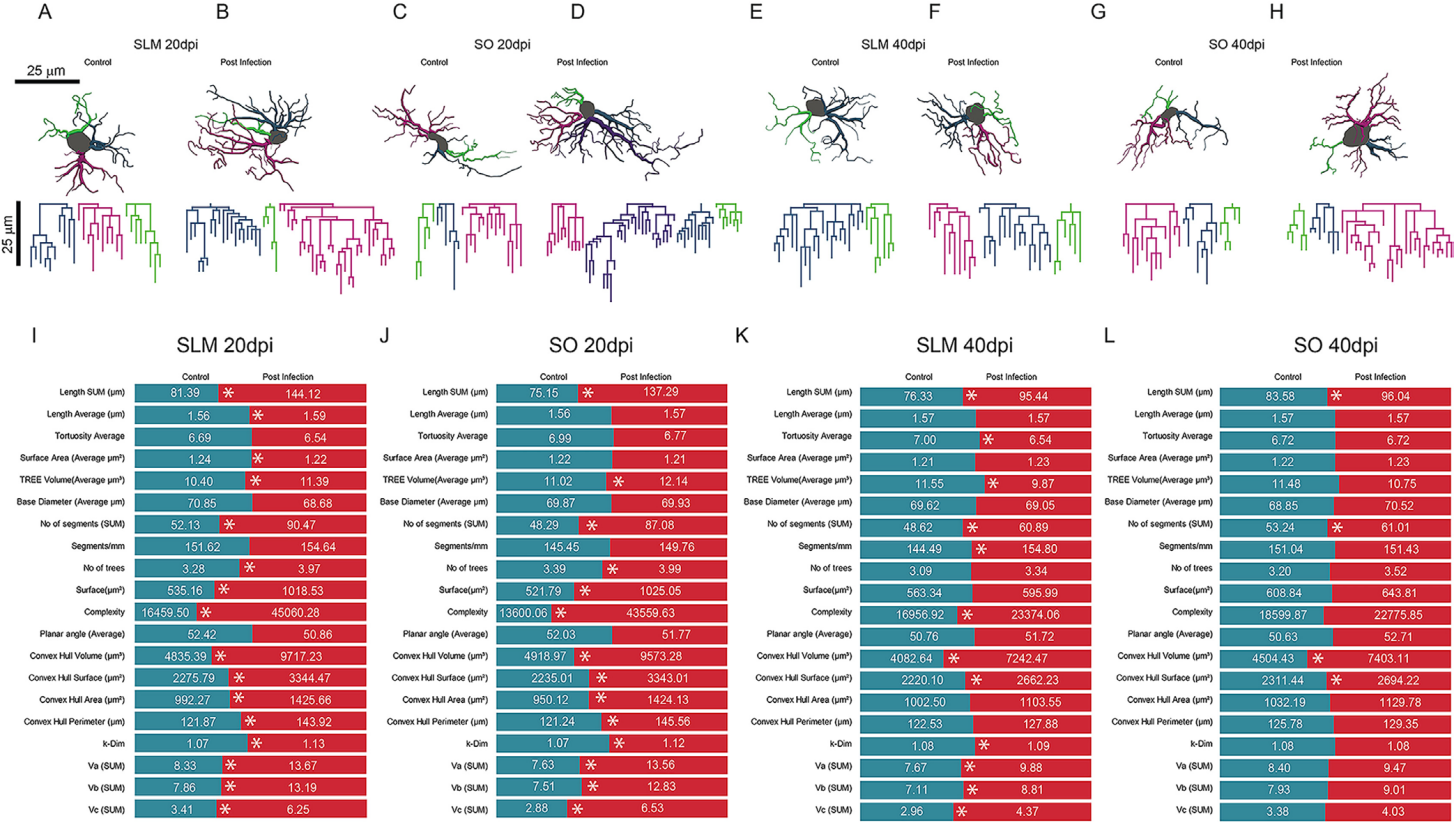
Morphometric alterations in astrocyte structure across hippocampal layers following viral infection. (A–H) Representative three‐dimensional reconstructions of GFAP‐positive astrocytes illustrate layer‐specific morphological changes in the *Stratum lacunosum‐moleculare* (SLM) and *Stratum oriens* (SO) at 20‐ and 40‐days post‐infection (dpi). (I–L) Quantitative comparisons of total process length, number of segments, surface area, and convex hull volume reveal significant increases in several parameters post‐infection, as indicated by *p*‐values from multivariate analyses. Each plot highlights the differential astrocytic response between cortical layers and time points, underscoring the dynamic and region‐specific nature of glial plasticity during encephalitis progression. A detailed description of the morphometric parameters is provided in Table S1. SLM astrocytes show the most pronounced changes, supporting the hypothesis of excitatory‐layer vulnerability to viral‐induced astrocytic remodeling.

In the SLM (Figure 4A,B,E,F), viral infection induced enhanced dendritic arborisation, reflected by increased tree volume, surface area, and convex hull perimeter. These increases were most pronounced at 20 dpi, suggesting early astrocytic hyperreactivity. While significant changes were also observed in the SO (Figure 4C,D,G,H), they were generally less marked than those seen in the SLM.

Summary plots in Figure 4I–L show statistically significant changes (*p* < 0.05) in most morphometric parameters. These results support the hypothesis that astrocyte structural plasticity differs across hippocampal layers and evolves over the course of viral encephalitis. Notably, SLM astrocytes exhibited the most substantial morphological alterations, consistent with the heightened vulnerability of excitatory regions to infection‐induced astrocytic remodeling.

### 3.2 Detailed Morphometric Analysis of GFAP‐Positive Astrocyte Processes in CA3 Hippocampus Associated With Viral Infection

**FIGURE 5:**
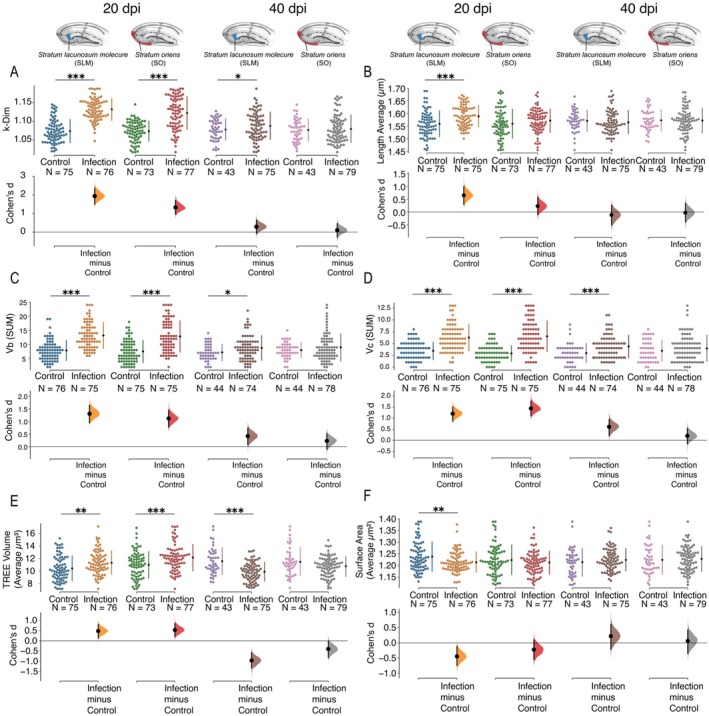
Three‐dimensional reconstruction and quantitative morphometric analysis of astrocytes from Swiss albino mice infected with the Piry virus. Cells were reconstructed in the *Stratum lacunosum‐moleculare* (SLM) and *Stratum oriens* (SO) of the hippocampus at two post‐infection time points: 20‐ and 40‐days post‐infection (dpi). (A–D) Graphs depict region‐ and time‐specific changes in morphometric parameters extracted from 3D reconstructions: (A) fractal dimension (k‐Dim), (B) average segment length (μm), (C) vertex type b (Vb), and (D) vertex type c (V). (E) mean tree volume (μm^3^), and (F) mean surface area (μm^2^). Lower panels display Cohen's *d* effect sizes, quantifying the magnitude of the differences between infected and control groups (Infection—Control). Asterisks denote statistical significance (*p < 0.05; **p < 0.01; ***p < 0.001). *N* refers to the number of cells analyzed per group. (E, F) Graphs depict region‐ and time‐specific changes in morphometric parameters extracted from 3D reconstructions: (E) TREE Volume (Average μm^3^) and (F) Surface Area (Average μm^3^). Lower panels display Cohen's *d* effect sizes, quantifying the magnitude of the differences between infected and control groups (Infection—Control). Asterisks denote statistical significance (*p < 0.05; **p < 0.01; ***p < 0.001). *N* refers to the number of cells analyzed per group.

To further examine astrocyte morphological complexity, we analyzed mean values and variability of morphometric features for SLM and SO astrocytes (Figure 5, Table S1). Effect sizes were calculated to quantify the magnitude of infection‐induced changes.

Fractal dimension (k‐Dim), a measure of morphological complexity, was significantly elevated in both layers at 20 dpi (Figure 5A). Infected animals showed increased k‐Dim relative to controls in the SLM (*t* = 11.61, df = 148.8, *p* < 0.001, *d* = 1.895) and SO (*t* = 8.81, df = 127.1, *p* < 0.001, *d* = 1.453). At 40 dpi, elevated k‐Dim persisted only in the SLM (*t* = 2.14, df = 107.6, *p* < 0.001, *d* = 0.396), with no significant differences in SO. Average segment length increased significantly in the SLM at 20 dpi (*t* = 4.21, df = 144.5, *p* < 0.001, *d* = 0.684) but returned to baseline by 40 dpi (Figure 5B). No significant changes were observed in the SO at either time point. Total branch length (vertex type b) was elevated in both layers at 20 dpi (SLM: *t* = 7.99, *p* < 0.001, *d* = 1.30; SO: *t* = 10.65, *p* < 0.001, *d* = 1.11), but remained elevated only in the SLM at 40 dpi (*t* = 2.52, *p* = 0.011, *d* = 0.46) (Figure 5C). Vertices type c (Vc) followed a similar pattern, with significant increases in both layers at 20 dpi (SLM: *t* = 7.35, *p* < 0.001, *d* = 1.19; SO: *t* = 8.78, *p* < 0.001, *d* = 1.43). By 40 dpi, only the SLM maintained elevated Vc counts (*t* = 3.50, *p* < 0.001, *d* = 0.64) (Figure 5D).

Astrocyte tree volume (TREE Volume) was significantly elevated at 20 dpi in both layers relative to controls (SLM: *t* = 3.17, df = 148.4, *p* = 0.002, *d* = 0.516; SO: *t* = 2.24, df = 148.0, *p* = 0.001, *d* = 0.529), with moderate effect sizes (Figure 5E, Table S3). At 40 dpi, only a significant reduction in cell volume was observed in the SLM (*t* = −4.70, df = 71.6, *p* < 0.001), with a large effect size (*d* = −0.924), indicating a possible late‐stage atrophy.

Finally, surface area analysis in SLM (Figure 5F, Table S4), but not in SO, revealed a discrete and transient response at 20 dpi (*t* = 2.62, df = 134.8, *p* = 0.01, *d* = 0.426). No significant changes were identified at 40 dpi.

Figures [Link], [Link] illustrate the differential morphological responses of GFAP‐positive astrocytes in SLM and SO of the CA3 hippocampal region following Piry virus infection, emphasizing additional morphometric parameters beyond those depicted in Figure 5. Figure S1 presents group comparisons for: (A) total branch length (SUM, μm), (B) number of segments, (C) surface area (μm^2^), and (D) convex hull volume (μm^3^), revealing structural remodeling consistent with reactive astrocytosis. Figure S2 focuses on spatial features of the convex hull, including: (E) convex hull surface (μm^2^), (F) convex hull area (μm^2^), (G) convex hull perimeter (μm), and (H) number of vertices (Va), highlighting modifications in the territory occupied by astrocytic processes. Figure S3 displays additional shape‐related parameters: (I) morphological complexity, (J) number of trees, (K) base diameter, and (L) average tortuosity. Figure S4 includes: (M) number of segments per millimeter, and (N) mean planar angle, further characterizing astrocyte arborization dynamics. Together, these Supporting Information figures reinforce the conclusion that astrocytes undergo distinct layer‐ and time‐dependent morphometric alterations during the progression of viral encephalitis.

A summary of the quantitative data used to generate Figures [Link], [Link] is provided in Table S4.

### 3.3 Astrocyte Morphotypes Identified by Hierarchical Clustering

Astrocytes exhibit layer‐specific morphological and functional specialization influenced by the local neurotransmitter environment (Karpf et al. 2022; Lanjakornsiripan et al. 2018). Reduced astrocyte morphological complexity in mice was shown to be associated with impaired cognitive behavior, raising the hypothesis that morphometric restoration may be therapeutically beneficial in disease (Endo et al. 2022). Astrocytes, which are the first cells infected by the virus crossing the blood–brain barrier, are highly diverse and brain‐region‐specific, changing their shapes when immunostimulated (Baldwin et al. 2024).

**FIGURE 6:**
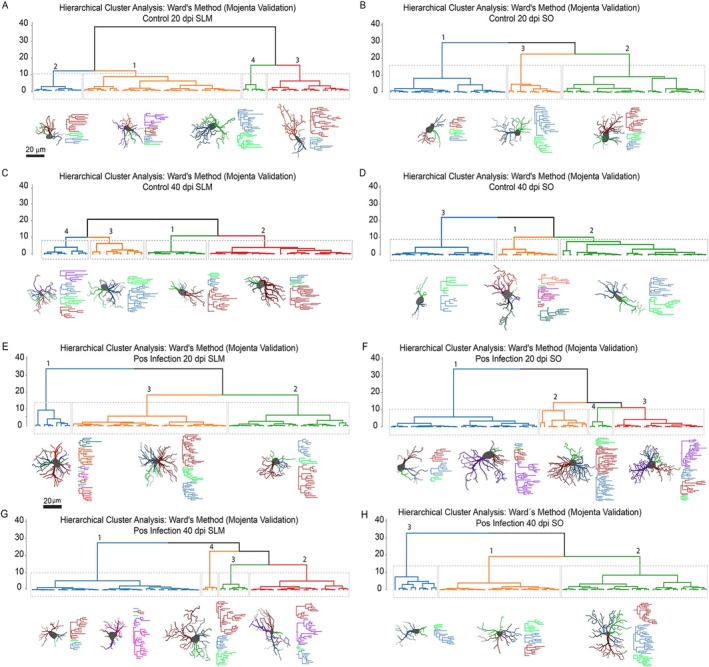
Comparison of the hierarchical clustering of astrocyte morphotypes in control and Piry virus‐infected animals. (A, B) Dendrograms and representative 3D‐reconstructed astrocytes from the *Stratum lacunosum‐moleculare* (SLM) and *Stratum oriens* (SO) at 20 dpi in controls and corresponding data in infected mice (C, D). The same was analyzed at 40 dpi in controls (E, F) and infected mice (G, H). Astrocytes were grouped using Ward's method with Euclidean distances, and representative cells were selected based on minimum Euclidean dissimilarity within each cluster. Each morphotype reflects distinct structural features such as process length, branching complexity, and convex hull volume. Viral infection increased morphotype heterogeneity, particularly in the SLM. Morphotypes exhibit reactive hypertrophy and increased branching complexity, indicating divergent responses to neuroinflammatory signaling.

In the present study, hierarchical cluster analysis using Ward's method revealed distinct astrocyte morphotypes for each experimental condition and hippocampal layer. In the SLM of control animals (Figure 6A,C), four morphotypes were identified at 20 and 40 dpi, showing moderate‐to‐high within‐group homogeneity. In contrast, the SO of control mice yielded three clusters at 20 and 40 dpi (Figure 6B,D), suggesting a slightly lower morphological astrocyte diversity in this layer than in SLM.

After infection, increased morphological complexity was noticed in both layers. Three and four morphotypes were observed in the SLM at 20 and 40 dpi respectively (Figure 6E,G). Higher heterogeneity was found in SO, mainly at 20 dpi, now with four morphotypes (Figure 6F), but less evident at 40 dpi, again with three subtypes (Figure 6H). Each dendrogram is accompanied by representative astrocytes from each cluster, illustrating distinctive branching patterns and spatial arrangements, indicating that viral infection induced divergent morphological adaptations within each hippocampal layer.

### 3.4 Cluster Validation and Discriminant Analysis

Linear discriminant analysis (LDA) consistently identified well‐defined clusters across all groups, with strong classification performance and significant separation metrics. As shown in Figure 6, discriminant functions effectively separated the clusters into all experimental groups and hippocampal layers. Notably, the SLM displayed greater group dispersion than the SO, consistent with higher morphometric complexity in this layer.

**FIGURE 7:**
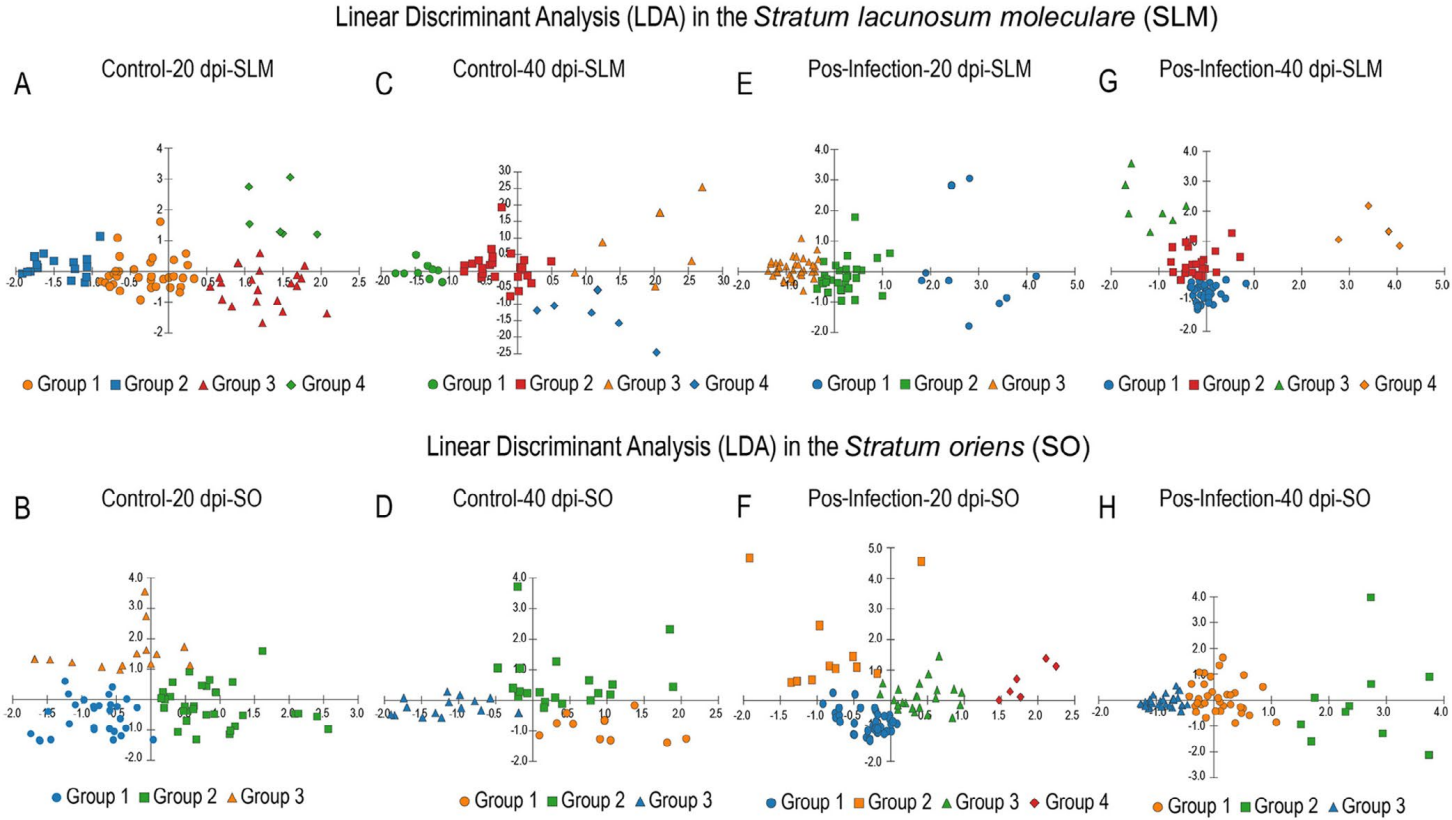
Linear discriminant analysis (LDA) applied to the morphometric characteristics of astrocytes across different experimental groups. The analysis was conducted in the *Stratum lacunosum‐moleculare* (SLM) (panels A, C, E, G) and *Stratum oriens* (SO) (panels B, D, F, H) regions of the hippocampus in mice, under control (20‐ and 40‐days post‐infection [dpi]) and post‐infection (20 and 40 dpi) conditions. Each point represents an individual astrocyte, grouped according to morphometric similarity. The different groups (1 to 4) are indicated by distinct shapes and colors.

In control animals (Figure 7A–D), clusters were well‐separated in both layers and time points, validating the initial hierarchical grouping. Following infection (Figure 7E–H), cluster overlap increased slightly, particularly in the SO at 40 dpi, reflecting more heterogeneous and potentially reactive astrocyte populations. Overall, LDA correctly classified astrocyte morphotypes with an accuracy exceeding 91%, confirming the discriminant power of the selected morphometric variables.

Linear discriminant analysis (LDA) consistently identified well‐defined clusters across all groups, with strong classification performance and significant separation metrics (Table 1). In control groups, classification accuracy ranged from 91.1% to 97.8%, with variance explained by canonical function 1 between 61.5% and 83.5%. Post‐infection groups generally showed higher classification rates (94.8%–98.7%) and, in several cases, function 1 accounted for over 99% of the variance, reflecting strong group separation.

**TABLE 1:** Summary of LDA results across groups.

Group	Clusters (*n*)	Accuracy (%)	Variance F1 (%)	Variance F2 (%)	Canonical corr F1	Canonical corr F2
Control 20 dpi SLM	4	94.7	83.48	16.52	0.93	0.75
Control 20 dpi SO	3	97.3	61.54	38.46	0.82	0.75
Control 40 dpi SLM	4	91.1	80.95	19.05	0.90	0.70
Control 40 dpi SO	3	97.8	69.85	30.15	0.82	0.68
Post‐infection 20 dpi SLM	3	97.3	99.27	0.73	0.94	—
Post‐infection 20 dpi SO	4	98.7	63.29	36.71	0.86	0.79
Post‐infection 40 dpi SLM	4	98.6	62.20	37.80	0.95	0.91
Post‐infection 40 dpi SO	4	94.8	99.32	0.68	0.93	0.20

Wilks' Lambda tests were highly significant in all comparisons (*p* < 0.001), confirming that both ZComplexity and ZConvex Hull Volume contributed meaningfully to group discrimination. Notably, in the post‐infection 40 dpi SO group, ZComplexity was most strongly associated with function 1 (standardized coefficient = 0.993), while ZConvex Hull Volume primarily contributed to function 2 (coefficient = 0.807). Tables [Link], [Link] show detailed information for each experimental group.

### 3.5 Astrocyte Complexity Across Layers and Time Points

**FIGURE 8:**
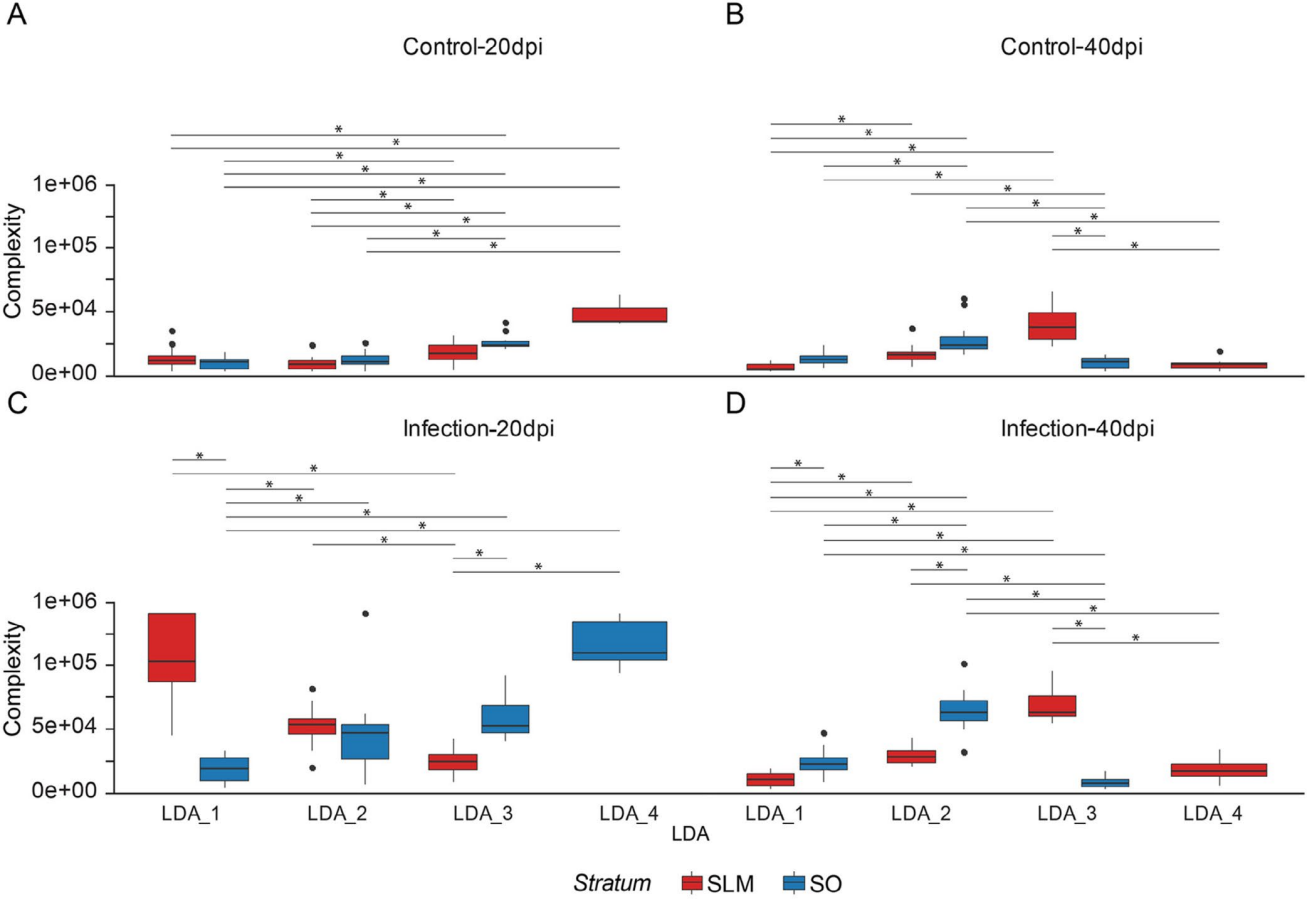
Comparative analysis of astrocyte complexity across conditions. Boxplots show the mean and distribution of LDA‐derived complexity scores for each cluster in control (A, B) and infected (C, D) groups. Astrocytes from infected animals exhibited significantly increased complexity, particularly in the SLM at 20 dpi. Differences between layers persisted at 40 dpi, with overlapping distributions indicating progressive morphological diversification.

Generalized linear models were applied to compare morphological complexity among clusters across layers and time points to quantify astrocytic reactivity further. Figure 8 presents a detailed comparative analysis of astrocyte morphological complexity in the SLM and SO of the hippocampal CA3 subfield, under both control and Piry virus‐infected conditions at 20 and 40 dpi. The boxplots are organized by Linear Discriminant Analysis (LDA) clusters and stratified by condition (control vs. infection) and time point (20 vs. 40 dpi). As shown in Figure 8, control animals exhibited significantly lower complexity in both layers at both 20 and 40 dpi (Figure 8A,B). In contrast, post‐infection groups exhibited markedly increased complexity, particularly in the SLM. At 20 dpi, complexity in the SLM of infected mice was significantly higher than in the SO (Figure 8C), suggesting early astrocytic reactivity in response to viral neuroinvasion. By 40 dpi (Figure 8D), the distinction between layers became more nuanced, with overlapping complexity distributions, but persistent statistical differences between several cluster combinations (LDA_1 through LDA_4), indicating a dynamic and persistent glial response across both layers. Infection induces layer‐specific and time‐dependent hypertrophic responses in astrocytes. At 20 dpi, SLM astrocytes (especially LDA_1) exhibit marked increases in complexity, possibly reflecting acute immune or metabolic reactivity. By 40 dpi, the pattern shifts, with some attenuation in SLM and persistent morphological changes in SO. The LDA‐based clustering suggests that infection not only alters morphology but may also induce astrocyte subpopulations with distinct structural phenotypes.

Figure 8 also illustrates significant differential alterations in astrocyte morphological complexity across the lacunosum‐molecular (SLM) and oriens (SO) strata of the CA3 hippocampal region in both control and Piry virus‐infected animals at 20 and 40 dpi. In control groups (panels A and B), morphological complexity remains relatively low and consistent across linear discriminant analysis (LDA)‐derived clusters (LDA_1 to LDA_4), with minimal variation observed between SLM (red) and SO (blue) astrocytes. In contrast, infected groups (panels C and D) exhibit a marked increase in astrocyte complexity, particularly in the SLM at 20 dpi (panel C), indicating an early and pronounced hypertrophic response within the excitatory microenvironment. SO astrocytes, in turn, demonstrate a more heterogeneous and delayed increase in complexity, especially evident at 40 dpi (panel D), suggestive of a more gradual and repressed reactivity within the inhibitory milieu. These findings highlight a layer‐specific and temporally dynamic astrocytic response to Piry virus infection, reflecting the distinct synaptic and functional architecture of the SLM and SO strata.

Table S13 summarizes the linear generalized model for the morphological complexity, including all experimental groups and time post‐infection. It provides pairwise comparisons of estimated marginal mean values for SLM and SO astrocytes.

### 3.6 Detailed Cluster Analysis by Group and Layer

We performed cluster analysis for each group to evaluate structural integrity and clustering quality, which is summarized in Table 2. Metrics included Hopkins statistics (clustering tendency), cophenetic correlation coefficient (hierarchical fit quality), and agglomeration coefficient (internal consistency).

**TABLE 2:** Cluster analysis metrics by group and hippocampal layer.

Group	Hopkins statistic	Cophenetic correlation	Agglomeration coefficient
Control 20 dpi SLM	0.924	0.656	0.976
Control 20 dpi SO	0.722	0.553	0.962
Control 40 dpi SLM	0.732	0.582	0.948
Control 40 dpi SO	0.643	0.482	0.949
Post‐infection 20 dpi SLM	0.934	0.803	0.984
Post‐infection 20 dpi SO	0.604	0.434	0.980
Post‐infection 40 dpi SLM	0.907	0.519	0.983
Post‐infection 40 dpi SO	0.676	0.725	0.979

For the control groups, the SLM at 20 dpi exhibited a strong clustering tendency (Hopkins 0.924) and the highest internal consistency (agglomeration coefficient 0.976), with moderate hierarchical fit (cophenetic 0.656). In contrast, the SO at 40 dpi showed a lower clustering tendency (Hopkins 0.643) and the weakest cophenetic correlation (0.482), though internal consistency remained high (agglomeration 0.949).

Post‐infection groups revealed marked clustering patterns, particularly in the 20 dpi SLM dataset (Hopkins 0.934; agglomeration 0.984) alongside the strongest hierarchical fit (cophenetic 0.803). The SO at 40 dpi also displayed a well‐fitted hierarchy (cophenetic 0.725), despite a moderate clustering tendency (Hopkins 0.676).

Together, these findings highlight robust clustering across datasets, with variation in hierarchical fit and clustering tendency reflecting biological diversity in astrocyte morphotypes concerning infection status and hippocampal layer.

## 4 Discussion

Our findings demonstrate that astrocytes in the CA3 hippocampal subfield undergo layer‐specific and temporally dynamic morphological changes following Piry virus infection. Early hypertrophic responses were more prominent in the SLM, whereas astrocytes in the SO exhibited a more gradual and heterogeneous profile of alterations, particularly evident at 40 days post‐infection (dpi). These observations are consistent with the notion that astrocytic reactivity is shaped by local microenvironmental factors and laminar identity (Khakh and Sofroniew 2015; Sofroniew 2020).

### 4.1 Layer‐Specific Patterns of Reactivity

Astrocytes in the SLM, a glutamate‐rich excitatory layer, displayed pronounced hypertrophy by 20 dpi, as reflected by increased branching complexity, tree volume, and convex hull parameters. Such alterations are consistent with enhanced glutamate clearance, increased metabolic demands, and neuroprotective buffering under excitotoxic stress. Indeed, morphological plasticity of this kind has been associated with the upregulation of glutamate transporters and neuroprotective support during neuroinflammation (Potokar et al. 2019; Tavčar et al. 2021). By contrast, SO astrocytes, which are embedded within a GABAergic‐rich environment, showed more restrained and delayed morphological responses. This muted reactivity may reflect the anti‐inflammatory influence of GABAergic signaling, known to suppress astrocytic pro‐inflammatory gene expression and to promote homeostatic phenotypes (Chen et al. 2023; Sofroniew 2020).

### 4.2 Morphotype Diversification Over Time

The transition from relatively homogeneous hypertrophy at 20 dpi to increased morphotype heterogeneity at 40 dpi suggests that astrocyte populations diversify adaptively under sustained inflammatory conditions. Previous work from our group using the same Piry virus model showed that viral replication within the CNS reaches its maximum at approximately 5 dpi and is followed by complete clearance from the brain parenchyma by 8 dpi, when viral antigens are no longer detectable (de Sousa et al. 2015; de Sousa et al. 2011). These observations indicate that the acute phase of infection is brief and that any subsequent cellular or structural alterations arise after the resolution of detectable viral presence.

The 20‐ and 40‐dpi intervals were therefore selected to examine astrocytic morphological adaptations during the post‐acute period, when infection is no longer active but secondary neuroinflammatory mechanisms remain engaged. These time points also correspond to a phase in which olfactory discrimination deficits have normalized, whereas hippocampal‐dependent behaviors, such as open‐field exploration and burrowing, show only partial recovery (de Sousa et al. 2015). Thus, both intervals provide a relevant window for assessing how sustained glial activation may influence hippocampal circuitry after viral clearance.

Focusing on these later stages allowed us to characterize long‐term astrocytic remodeling associated with persistent neuroinflammation and incomplete behavioral recovery. This approach provides insight into the lasting effects of viral encephalitis on hippocampal structure and function, rather than the transient changes linked to the acute infectious phase.

Cluster analysis revealed multiple morphotypes, echoing patterns observed in other viral encephalitis models (Davé and Klein 2023; Dos Santos et al. 2025; Liddelow and Barres 2017). Although these morphotypes may appear consistent with neuroprotective or maladaptive features, such functional attributions remain speculative. We therefore interpret these clusters as indicators of morphological diversity, without implying direct functional roles. Future studies using calcium imaging, single‐cell transcriptomics, or targeted manipulation of astrocytic pathways will be necessary to clarify whether distinct morphotypes correspond to divergent biological functions.

### 4.3 Observational Nature and Alternative Mechanisms

While it is tempting to attribute the divergent astrocytic responses directly to excitatory versus inhibitory inputs, our study is strictly observational and does not establish causality. Multiple factors—such as neuronal activity, microglial activation, hypoxia, or cell death—may also contribute to the observed differences. Integrating morphometric analyses with synaptic markers (e.g., VGLUT, PSD95, VGAT) or functional assays will be critical to test whether neurotransmitter context directly shapes astrocytic morphology in this model. The selection of the *Stratum lacunosum‐moleculare* (SLM) and *Stratum oriens* (SO) of CA3 was guided by evidence that astrocyte morphology and physiology exhibit strong laminar specificity within the hippocampus. Astrocytes differ across sublayers in their transcriptional programs, metabolic profiles, process architecture, and functional engagement with local circuits, supporting the broader view that they contribute to regionally specialized forms of synaptic and homeostatic regulation (Khakh and Sofroniew 2015; Bayraktar et al. 2020; Miller et al. 2019). Examining astrocytes in distinct CA3 sublayers therefore provides an opportunity to resolve how layer‐dependent cellular phenotypes shape vulnerability and response to inflammatory challenges.

Our previous work using the Piry virus model also supported the strategic focus on these sublayers. During the course of infection, viral antigens are not detected in the CA3 SO or SLM, whereas neighboring regions, particularly the dentate gyrus, continue to exhibit robust immune reactivity (de Sousa et al. 2011). This restricted antigen distribution suggests that CA3 mounts a more transient innate immune response, creating a relatively stable microenvironment in which subtle, progressive astrocytic changes can be detected without the confounding influence of ongoing viral replication.

In addition, astrocytes in SLM and SO are known to support distinct physiological roles, particularly in synaptic modulation, calcium dynamics, and integration of extrahippocampal versus intrahippocampal inputs (Matyash and Kettenmann 2010; Sofroniew and Vinters 2010; Bayraktar et al. 2020). SLM astrocytes interact with perforant‐path terminals from the entorhinal cortex, whereas SO astrocytes participate in the regulation of local recurrent collaterals and inhibitory networks. Evaluating both sublayers thus allowed us to determine whether viral encephalitis induces convergent or divergent morphological adaptations across functionally distinct astrocyte populations.

Finally, all astrocytes were sampled from the same segment of the septotemporal axis to maintain anatomical consistency and avoid positional variability as a confounding factor. This approach ensured that observed differences reflected layer‐specific responses rather than longitudinal heterogeneity along the CA3 axis.

Interestingly, the apparent reduction in morphotype diversity in the SLM at 20 dpi may represent a convergence of astrocytic states under acute inflammatory stress, whereas the greater heterogeneity observed at 40 dpi suggests a progressive diversification of responses over time. These dynamics align with evidence of astrocyte heterogeneity and plasticity in CNS disease (Baldwin et al. 2024; Liddelow and Barres 2017).

### 4.4 Methodological Strengths and Limitations

The combined use of hierarchical clustering and linear discriminant analysis (LDA) provided robust morphotype classification based on multiple structural parameters, such as convex hull volume, branching complexity, and tree volume, offering greater resolution than GFAP intensity alone. This approach is in line with previous work emphasizing structural metrics in astroglial phenotyping (Baldwin et al. 2024; Colombo and Farina 2016; Delgado‐García et al. 2024). Morphotype dispersion was particularly pronounced in the SLM, highlighting its higher morphological plasticity and suggesting greater susceptibility to infection‐related stress (Zhang et al. 2024).

Nonetheless, several limitations should be acknowledged. First, this study included only female Swiss mice, which restricts generalisability across sexes and strains. Sex‐dependent differences in astrocytic reactivity have been described, with distinct transcriptional and morphological signatures (Gozlan et al. 2024). However, this choice was deliberate and grounded in the need to minimize confounding neuroinflammatory influences associated with stress physiology. In our animal colony, the presence of males reliably increases territorial aggression, dominance‐hierarchy formation, and repetitive stress‐related behaviors, all of which elevate circulating corticosterone levels. Given the high density of glucocorticoid receptors in the hippocampus (Sapolsky et al. 1985), these stress‐mediated endocrine changes can independently modify astrocyte morphology and glial activation. Such alterations would introduce variability that is unrelated to the viral challenge itself.

Under these conditions, including males would add a second, and uncontrolled, driver of astrocytic remodeling, complicating the interpretation of infection‐specific morphometric effects (McEwen and Morrison 2013; Bartolomucci et al. 2001). To maintain experimental consistency and reduce variance attributable to stress reactivity, we therefore conducted all analyses in female mice (Koolhaas et al. 2011). This strategy enabled a clearer assessment of virus‐induced astrocytic changes by avoiding confounding morphological modulation driven by male‐specific social stress.

Second, GFAP staining, although widely used, does not capture the full extent of fine astrocytic processes. Broader markers such as Aldh1L1 could improve future morphological resolution (Baldwin et al. 2024). Finally, although morphotypes were robustly defined, their functional correlates remain unresolved. Advanced techniques, including calcium imaging or single‐cell omics (Batiuk et al. 2020; de Ceglia et al. 2023) will be required to link morphology with physiological outcomes.

Although the present study provides a detailed three‐dimensional morphometric analysis of astrocytes, it is important to acknowledge the limitation inherent to the use of anti‐glial fibrillary acidic protein (GFAP) immunolabeling as the sole morphological marker. GFAP primarily highlights the intermediate filament network within the main astrocytic branches and soma, but it does not fully capture the extensive fine processes that form the perisynaptic astrocytic domain (Bushong et al. 2004). These thin peripheral processes, which are largely devoid of GFAP filaments, play critical roles in neurotransmitter uptake, synaptic modulation, and metabolic coupling with neurons. Consequently, while the present reconstructions reliably represent the core structural framework of reactive astrocytes, they may underestimate the full spatial complexity of the astrocytic arbor, particularly under conditions of mild activation or remodeling. Future studies combining GFAP labeling with cytoplasmic or membrane‐filling markers (such as Aldh1l1, S100β, or genetically encoded fluorescent reporters) will help to more completely characterize the astrocytic morphological response to viral encephalitis.

### 4.5 Broader Implications of the Piry Virus Model

The Piry virus model proved valuable for this study, given its consistent tropism for the rodent limbic system and capacity to elicit hippocampal inflammation (da Silva Creão et al. 2021; de Sousa et al. 2015).

Although the virus induces only mild symptoms in humans, its neuroinvasiveness enables systematic exploration of glial responses in well‐defined hippocampal subregions, thereby offering an informative platform for understanding astrocyte plasticity during encephalitis.

## 5 Conclusion

In summary, this work provides a detailed morphometric description of astrocyte responses to viral encephalitis across hippocampal layers. The results highlight both the diversity and plasticity of astrocytic morphology and underscore the importance of the local microenvironment in shaping glial reactivity. By presenting these findings within an observational framework, the study establishes a foundation for future mechanistic investigations aimed at understanding how astrocyte heterogeneity influences the course and outcomes of viral CNS infections.

## Conflicts of Interest

The authors declare no conflicts of interest.

## Supporting information


**Figure S1:** Three‐dimensional reconstruction of astrocytes from Swiss albino mice infected with Piry virus in the *Stratum lacunosum‐moleculare* (SLM) and *Stratum oriens* (SO) layers, at 20‐ and 40‐days post‐infection (dpi). The graphs represent morphological measures extracted from the 3D reconstructions: (A) total branch length (SUM, μm), (B) number of segments, (C) surface area (μm^2^) and (D) convex hull volume (μm^3^). The lower graphs show the effect sizes (Cohen's *d*), highlighting the magnitude of the differences between the groups (Infection—Control). The analyses show regional and temporal morphological changes in astrocytes in response to viral infection. *N* indicates the number of cells demonstrated per group. Asterisks indicate statistically significant differences (*p < 0.05; **p < 0.01; ***p < 0.001).

**Figure S2:** Three‐dimensional reconstruction of astrocytes from Swiss albino mice infected with Piry virus in the *Stratum lacunosum‐moleculare* (SLM) and *Stratum oriens* (SO) layers, in the time windows of 20‐ and 40‐days post‐infection (dpi). The graphs represent morphological measures extracted from the 3D reconstructions: (E) Convex Hull Surface (μm^2^), (F) Convex Hull Area (μm^2^), (G) Convex Hull Perimeter (μm^2^), (H) Vertex A (Va (SUM)). The lower graphs show the effect sizes (Cohen's *d*), highlighting the magnitude of the differences between the groups (Infection—Control). The analyses demonstrated regional and temporal morphological changes in astrocytes in response to viral infection. *N* indicates the number of cells analyzed per group. Asterisks indicate statistically significant differences (*p < 0.05; **p < 0.01; ***p < 0.001).

**Figure S3:** Three‐dimensional reconstruction of astrocytes from Swiss albino mice infected with Piry virus in the *Stratum lacunosum‐moleculare* (SLM) and *Stratum oriens* (SO) layers, in the temporal windows of 20‐ and 40‐days post‐infection (dpi). The graphs represent morphological measures extracted from the 3D reconstructions: morphological complexity (I), number of trees (J), base diameter (K) and mean tortuosity (L). The lower graphs show the effect sizes (Cohen's *d*), highlighting the magnitude of the differences between the groups (Infection—Control). The analyses demonstrated regional and temporal morphological changes in astrocytes in response to viral infection. *N* indicates the number of cells analyzed per group. Asterisks indicate statistically significant differences (*p < 0.05; **p < 0.01; ***p < 0.001).

**Figure S4:** Three‐dimensional reconstruction of astrocytes from Swiss albino mice infected with Piry virus in the *Stratum lacunosum‐moleculare* (SLM) and *Stratum oriens* (SO) layers, at 20‐ and 40‐days post‐infection (dpi). The graphs represent morphological measures extracted from the 3D reconstructions: (M) segments/mm and (N) planar angle (average). The lower graphs show the effect sizes (Cohen's *d*), highlighting the magnitude of the differences between the groups (Infection—Control). The analyses demonstrated regional and temporal morphological changes in astrocytes in response to viral infection. *N* indicates the number of cells analyzed per group. Asterisks indicate statistically significant differences (*p < 0.05; **p < 0.01; ***p < 0.001).

**Table S1:** Morphometric parameters of three‐dimensional reconstructed astrocytes.

**Table S2:** Summary of multivariate statistical procedures.

**Table S3:** Three‐dimensional morphometric analysis of astrocytes from *Swiss albino* mice infected with the Piry virus, reconstructed in the stratum lacunosum moleculare (SLM) and stratum oriens (SO) regions at two post‐infection time points: 20 and 40 days (dpi). Data are presented as mean ± standard deviation, and comparisons between control and infected groups were performed using Welch's corrected Student's *t*‐test for unequal variances. The table also provides *t*‐values, degrees of freedom (df), *p*‐values, and effect sizes (Cohen's *d*). *p*‐values < 0.05 were considered statistically significant.

**Table S4:** Three‐dimensional morphometric analysis of astrocytes from *Swiss albino* mice infected with the Piry virus, reconstructed in the stratum lacunosum moleculare (SLM) and stratum oriens (SO) regions at two post‐infection time points: 20 and 40 days (dpi). Data are presented as mean ± standard deviation, and comparisons between control and infected groups were performed using Welch's corrected Student's *t*‐test for unequal variances. The table also provides *t*‐values, degrees of freedom (df), *p*‐values, and effect sizes (Cohen's *d*). *p*‐values < 0.05 were considered statistically significant.

**Table S5:** Discriminant analysis results for the control 20 dpi SO group.

**Table S6:** Discriminant analysis results for the control 40 dpi SLM group.

**Table S7:** Discriminant analysis results for the control 40 dpi SO group.

**Table S8:** Discriminant analysis results for the post‐infection 20 dpi SLM group.

**Table S9:** Discriminant analysis results for the post‐infection 20 dpi SO group.

**Table S10:** Discriminant analysis results for the post‐infection 40 dpi SLM group.

**Table S11:** Discriminant analysis results for the post‐infection 40 dpi SO group.

**Table S12:** Hierarchical cluster analyses by experimental group.

**Table S13:** Summary of multivariate statistical steps.

## Data Availability

The data that support the findings of this study are available from the corresponding author upon reasonable request.

## References

[hipo70085-bib-0002] Baldwin, K. T. , K. K. Murai , and B. S. Khakh . 2024. “Astrocyte Morphology.” Trends in Cell Biology 34, no. 7: 547–565. 10.1016/j.tcb.2023.09.006. 38180380 PMC11590062

[hipo70085-bib-0057] Bartolomucci, A. , P. Palanza , L. Gaspani , et al. 2001. “Social Status in Mice: Behavioral, Endocrine and Immune Changes Are Context Dependent.” Physiology & Behavior 73, no. 3: 401–410. 11438368 10.1016/s0031-9384(01)00453-x

[hipo70085-bib-0003] Batiuk, M. Y. , A. Martirosyan , J. Wahis , et al. 2020. “Identification of Region‐Specific Astrocyte Subtypes at Single Cell Resolution.” Nature Communications 11, no. 1: 1220. 10.1038/s41467-019-14198-8. PMC7058027 32139688

[hipo70085-bib-0051] Bayraktar, O. A. , T. Bartels , S. Holmqvist , et al. 2020. “Astrocyte Layers in the Mammalian Cerebral Cortex Revealed by a Single‐Cell in Situ Transcriptomic Map.” Nature Neuroscience 23, no. 4: 500–509. 32203496 10.1038/s41593-020-0602-1 PMC7116562

[hipo70085-bib-0004] Bohmwald, K. , C. A. Andrade , N. M. S. Gálvez , V. P. Mora , J. T. Muñoz , and A. M. Kalergis . 2021. “The Causes and Long‐Term Consequences of Viral Encephalitis.” Frontiers in Cellular Neuroscience 15: 755875. 10.3389/fncel.2021.755875. 34916908 PMC8668867

[hipo70085-bib-0005] Boulton, M. , and A. Al‐Rubaie . 2025. “Neuroinflammation and Neurodegeneration Following Traumatic Brain Injuries.” Anatomical Science International 100, no. 1: 3–14. 10.1007/s12565-024-00778-2. 38739360 PMC11725545

[hipo70085-bib-0006] Braga, M. , and Z. Santos . 2008. “Neurotropismo e neuropatologia secundárias à infecção experimental por espécies virais selecionadas da família Rhabdoviridae e cinética da infecção pelo arbovírus Piry em modelo murino.” Trabalho de Conclusão de Curso/Graduação Médica, Federal Universiy of Pará, Belém.

[hipo70085-bib-0007] Bushong, E. A. , M. E. Martone , and M. H. Ellisman . 2004. “Maturation of Astrocyte Morphology and the Establishment of Astrocyte Domains During Postnatal Hippocampal Development.” International Journal of Developmental Neuroscience 22, no. 2: 73–86. 10.1016/j.ijdevneu.2003.12.008. 15036382

[hipo70085-bib-0008] Carlo, C. N. , and C. F. Stevens . 2011. “Analysis of Differential Shrinkage in Frozen Brain Sections and Its Implications for the Use of Guard Zones in Stereology.” Journal of Comparative Neurology 519, no. 14: 2803–2810. 10.1002/cne.22652. 21491430

[hipo70085-bib-0009] Chen, W. , J. M. Gullett , R. E. Tweedell , and T. D. Kanneganti . 2023. “Innate Immune Inflammatory Cell Death: PANoptosis and PANoptosomes in Host Defense and Disease.” European Journal of Immunology 53, no. 11: e2250235. 10.1002/eji.202250235. 36782083 PMC10423303

[hipo70085-bib-0010] Colombo, E. , and C. Farina . 2016. “Astrocytes: Key Regulators of Neuroinflammation.” Trends in Immunology 37, no. 9: 608–620. 10.1016/j.it.2016.06.006. 27443914

[hipo70085-bib-0011] da Silva Creão, L. S. , J. B. T. Neto , C. M. de Lima , et al. 2021. “Microglial Metamorphosis in Three Dimensions in Virus Limbic Encephalitis: An Unbiased Pictorial Representation Based on a Stereological Sampling Approach of Surveillant and Reactive Microglia.” Brain Sciences 11, no. 8: 1009. 10.3390/brainsci11081009. 34439628 PMC8393838

[hipo70085-bib-0012] Davé, V. A. , and R. S. Klein . 2023. “The Multitaskers of the Brain: Glial Responses to Viral Infections and Associated Post‐Infectious Neurologic Sequelae.” Glia 71, no. 4: 803–818. 10.1002/glia.24294. 36334073 PMC9931640

[hipo70085-bib-0013] de Ceglia, R. , A. Ledonne , D. G. Litvin , et al. 2023. “Specialized Astrocytes Mediate Glutamatergic Gliotransmission in the CNS.” Nature 622, no. 7981: 120–129. 10.1038/s41586-023-06502-w. 37674083 PMC10550825

[hipo70085-bib-0014] de Sousa, A. A. , R. R. Dos Reis , C. M. de Lima , et al. 2015. “Three‐Dimensional Morphometric Analysis of Microglial Changes in a Mouse Model of Virus Encephalitis: Age and Environmental Influences.” European Journal of Neuroscience 42, no. 4: 2036–2050. 10.1111/ejn.12951. 25980955

[hipo70085-bib-0015] Delgado‐García, L. M. , A. C. Ojalvo‐Sanz , T. K. E. Nakamura , E. Martín‐López , M. Porcionatto , and L. Lopez‐Mascaraque . 2024. “Dissecting Reactive Astrocyte Responses: Lineage Tracing and Morphology‐Based Clustering.” Biological Research 57, no. 1: 54. 10.1186/s40659-024-00532-y. 39143594 PMC11323641

[hipo70085-bib-0016] DOE . 2023. “Multivariate Analysis (R Package Version 2.1.4). R Package for Multivariate Exploration, Clustering, and LDA Analysis.” https://cran.r‐project.org.

[hipo70085-bib-0017] Dos Santos, A. S. , M. G. da Costa , W. de Almeida , et al. 2025. “Long‐Term Impact of Congenital Zika Virus Infection on the Rat Hippocampus: Neuroinflammatory, Glial Alterations and Sex‐Specific Effects.” Brain Research 1850: 149421. 10.1016/j.brainres.2024.149421. 39710052

[hipo70085-bib-0018] Endo, F. , A. Kasai , J. S. Soto , et al. 2022. “Molecular Basis of Astrocyte Diversity and Morphology Across the CNS in Health and Disease.” Science 378, no. 6619: eadc9020. 10.1126/science.adc9020. 36378959 PMC9873482

[hipo70085-bib-0020] Finch, W. , and B. French . 2015. Latent Variable Modeling With R. Routledge.

[hipo70085-bib-0021] Freund, T. F. , and M. Antal . 1988. “GABA‐Containing Neurons in the Septum Control Inhibitory Interneurons in the Hippocampus.” Nature 336, no. 6195: 170–173. 10.1038/336170a0. 3185735

[hipo70085-bib-0022] Gozlan, E. , Y. Lewit‐Cohen , and D. Frenkel . 2024. “Sex Differences in Astrocyte Activity.” Cells 13, no. 20: 1724. 10.3390/cells13201724. 39451242 PMC11506538

[hipo70085-bib-0023] Hair, J. , W. Black , B. Babin , and R. Anderson . 2014. Multivariate Data Analysis. 7th ed. Pearson Education Limited.

[hipo70085-bib-0024] Hosseini, S. , and M. Korte . 2023. “How Viral Infections Cause Neuronal Dysfunction: A Focus on the Role of Microglia and Astrocytes.” Biochemical Society Transactions 51, no. 1: 259–274. 10.1042/BST20220771. 36606670

[hipo70085-bib-0025] Karpf, J. , P. Unichenko , N. Chalmers , et al. 2022. “Dentate Gyrus Astrocytes Exhibit Layer‐Specific Molecular, Morphological and Physiological Features.” Nature Neuroscience 25, no. 12: 1626–1638. 10.1038/s41593-022-01192-5. 36443610

[hipo70085-bib-0026] Kaur, G. , P. Pant , R. Bhagat , and P. Seth . 2023. “Zika Virus E Protein Modulates Functions of Human Brain Microvascular Endothelial Cells and Astrocytes: Implications on Blood‐Brain Barrier Properties.” Frontiers in Cellular Neuroscience 17: 1173120. 10.3389/fncel.2023.1173120. 37545876 PMC10399241

[hipo70085-bib-0027] Khakh, B. S. , and M. V. Sofroniew . 2015. “Diversity of Astrocyte Functions and Phenotypes in Neural Circuits.” Nature Neuroscience 18, no. 7: 942–952. 10.1038/nn.4043. 26108722 PMC5258184

[hipo70085-bib-0058] Koolhaas, J. M. , A. Bartolomucci , B. Buwalda , et al. 2011. “Stress Revisited: A Critical Evaluation of the Stress Concept.” Neuroscience & Biobehavioral Reviews 35, no. 5: 1291–1301. 21316391 10.1016/j.neubiorev.2011.02.003

[hipo70085-bib-0028] Lanjakornsiripan, D. , B. J. Pior , D. Kawaguchi , et al. 2018. “Layer‐Specific Morphological and Molecular Differences in Neocortical Astrocytes and Their Dependence on Neuronal Layers.” Nature Communications 9, no. 1: 1623. 10.1038/s41467-018-03940-3. PMC5915416 29691400

[hipo70085-bib-0029] Li, L. , C. Acioglu , R. F. Heary , and S. Elkabes . 2021. “Role of Astroglial Toll‐Like Receptors (TLRs) in Central Nervous System Infections, Injury and Neurodegenerative Diseases.” Brain, Behavior, and Immunity 91: 740–755. 10.1016/j.bbi.2020.10.007. 33039660 PMC7543714

[hipo70085-bib-0030] Liddelow, S. A. , and B. A. Barres . 2017. “Reactive Astrocytes: Production, Function, and Therapeutic Potential.” Immunity 46, no. 6: 957–967. 10.1016/j.immuni.2017.06.006. 28636962

[hipo70085-bib-0031] Maechler, M. , P. Rousseeuw , A. Struyf , M. Hubert , and K. Hornik . 2023. “Cluster: Cluster Analysis Basics and Extensions (Version 2.1.4).” R Package.

[hipo70085-bib-0053] Matyash, V. , and H. Kettenmann . 2010. “Heterogeneity in Astrocyte Morphology and Physiology.” Brain Research Reviews 63, no. 1‐2: 2–10. 20005253 10.1016/j.brainresrev.2009.12.001

[hipo70085-bib-0056] McEwen, B. S. , and J. H. Morrison . 2013. “The Brain on Stress: Vulnerability and Plasticity of the Prefrontal Cortex Over the Life Course.” Neuron 79, no. 1: 16–29. 23849196 10.1016/j.neuron.2013.06.028 PMC3753223

[hipo70085-bib-0032] Megías, M. , Z. Emri , T. F. Freund , and A. I. Gulyás . 2001. “Total Number and Distribution of Inhibitory and Excitatory Synapses on Hippocampal CA1 Pyramidal Cells.” Neuroscience 102, no. 3: 527–540. 10.1016/s0306-4522(00)00496-6. 11226691

[hipo70085-bib-0033] Mielcarska, M. B. , K. Skowrońska , Z. Wyżewski , and F. N. Toka . 2021. “Disrupting Neurons and Glial Cells Oneness in the Brain—The Possible Causal Role of Herpes Simplex Virus Type 1 (HSV‐1) in Alzheimer's Disease.” International Journal of Molecular Sciences 23, no. 1: 242. 10.3390/ijms23010242. 35008671 PMC8745046

[hipo70085-bib-0052] Miller, S. J. , T. Philips , N. Kim , et al. 2019. “Molecularly Defined Cortical Astroglia Subpopulation Modulates Neurons via Secretion of Norrin.” Nature Neuroscience 22, no. 5: 741–752. 30936556 10.1038/s41593-019-0366-7 PMC6551209

[hipo70085-bib-0034] Mojena, R. 1977. “Hierarchical Grouping Methods and Stopping Rules: An Evaluation.” Computer Journal 20: 359–363.

[hipo70085-bib-0035] Mora, V. P. , A. M. Kalergis , and K. Bohmwald . 2024. “Neurological Impact of Respiratory Viruses: Insights Into Glial Cell Responses in the Central Nervous System.” Microorganisms 12, no. 8: 1713. 10.3390/microorganisms12081713. 39203555 PMC11356956

[hipo70085-bib-0036] Nourbakhsh, F. , M. I. Read , G. E. Barreto , and A. Sahebkar . 2021. “Astrocytes and Inflammasome: A Possible Crosstalk in Neurological Diseases.” Current Medicinal Chemistry 28, no. 24: 4972–4994. 10.2174/0929867328666210301105422. 33645473

[hipo70085-bib-0037] Pavlou, A. , F. Mulenge , O. L. Gern , et al. 2024. “Orchestration of Antiviral Responses Within the Infected Central Nervous System.” Cellular & Molecular Immunology 21, no. 9: 943–958. 10.1038/s41423-024-01181-7. 38997413 PMC11364666

[hipo70085-bib-0038] Paxinos, G. , and K. Franklin . 2001. The Mouse Brain in Stereotaxic Coordinates. Academic Press.

[hipo70085-bib-0040] Potokar, M. , J. Jorgačevski , and R. Zorec . 2019. “Astrocytes in Flavivirus Infections.” International Journal of Molecular Sciences 20, no. 3: 691. 10.3390/ijms20030691. PMC6386967 30736273

[hipo70085-bib-0055] Sapolsky, R. M. , L. C. Krey , and B. S. McEWEN . 1985. “Prolonged Glucocorticoid Exposure Reduces Hippocampal Neuron Number: Implications for Aging.” Journal of Neuroscience 5, no. 5: 1222–1227. 3998818 10.1523/JNEUROSCI.05-05-01222.1985 PMC6565052

[hipo70085-bib-0041] Sofroniew, M. V. 2020. “Astrocyte Reactivity: Subtypes, States, and Functions in CNS Innate Immunity.” Trends in Immunology 41, no. 9: 758–770. 10.1016/j.it.2020.07.004. 32819810 PMC7484257

[hipo70085-bib-0054] Sofroniew, M. V. , and H. V. Vinters . 2010. “Astrocytes: Biology and Pathology.” Acta Neuropathologica 119, no. 1: 7–35. 20012068 10.1007/s00401-009-0619-8 PMC2799634

[hipo70085-bib-0042] Soung, A. , and R. S. Klein . 2018. “Viral Encephalitis and Neurologic Diseases: Focus on Astrocytes.” Trends in Molecular Medicine 24, no. 11: 950–962. 10.1016/j.molmed.2018.09.001. 30314877 PMC6546292

[hipo70085-bib-0050] Sousa, D. , A. Andrade , R. Reis , et al. 2011. “Influence of Enriched Environment on Viral Encephalitis Outcomes: Behavioral and Neuropathological Changes in Albino Swiss Mice.” PLoS One 6, no. 1: e15597. 21264301 10.1371/journal.pone.0015597 PMC3019164

[hipo70085-bib-0043] Steardo, L. , and C. Scuderi . 2023. “Astrocytes and the Psychiatric Sequelae of COVID‐19: What we Learned From the Pandemic.” Neurochemical Research 48, no. 4: 1015–1025. 10.1007/s11064-022-03709-7. 35922744 PMC9362636

[hipo70085-bib-0044] Tabachnick, B. , and L. Fidell . 2013. Using Multivariate Statistics. 6th ed. Pearson.

[hipo70085-bib-0045] Tavčar, P. , M. Potokar , M. Kolenc , et al. 2021. “Neurotropic Viruses, Astrocytes, and COVID‐19.” Frontiers in Cellular Neuroscience 15: 662578. 10.3389/fncel.2021.662578. 33897376 PMC8062881

[hipo70085-bib-0046] Tzilivaki, A. , J. J. Tukker , N. Maier , P. Poirazi , R. P. Sammons , and D. Schmitz . 2023. “Hippocampal GABAergic Interneurons and Memory.” Neuron 111, no. 20: 3154–3175. 10.1016/j.neuron.2023.06.016. 37467748 PMC10593603

[hipo70085-bib-0047] Venkatesan, A. , B. D. Michael , J. C. Probasco , R. G. Geocadin , and T. Solomon . 2019. “Acute Encephalitis in Immunocompetent Adults.” Lancet 393, no. 10172: 702–716. 10.1016/S0140-6736(18)32526-1. 30782344

[hipo70085-bib-0049] Zhang, Y. , Z. Wang , F. Xu , et al. 2024. “Progress of Astrocyte‐Neuron Crosstalk in Central Nervous System Diseases.” Neurochemical Research 49, no. 12: 3187–3207. 10.1007/s11064-024-04241-6. 39292330

