# Supplementary figures and images for: Layer‐Specific Astrocyte Morphological Responses in the CA3 Hippocampus Region During Piry Virus‐Induced Encephalitis
Source: Hippocampus. 2026 Feb 22;36(2):e70085. doi: 10.1002/hipo.70085 (PMC12926523; doi:10.1002/hipo.70085)

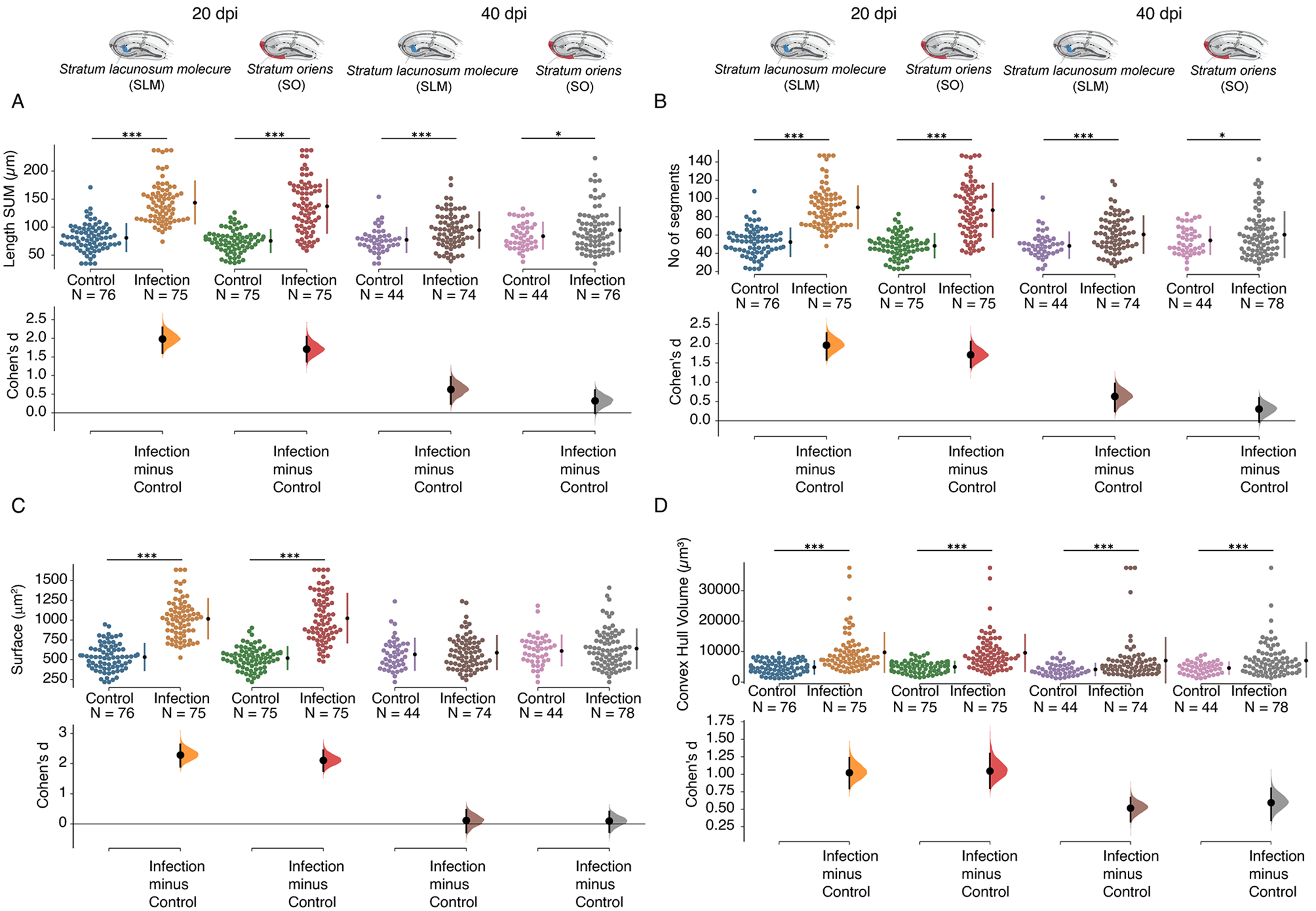

Supplement: Supplementary file 1 — Figure S1: Three‐dimensional reconstruction of astrocytes from Swiss albino mice infected with Piry virus in the Stratum lacunosum‐moleculare (SLM) and Stratum oriens (SO) layers, at 20‐ and 40‐days post‐infection (dpi). The graphs represent morphological measures extracted from the 3D reconstructions: (A) total branch length (SUM, μm), (B) number of segments, (C) surface area (μm2) and (D) convex hull volume (μm3). The lower graphs show the effect sizes (Cohen's d), highlighting the magnitude of the differences between the groups (Infection—Control). The analyses show regional and temporal morphological changes in astrocytes in response to viral infection. N indicates the number of cells demonstrated per group. Asterisks indicate statistically significant differences (*p < 0.05; **p < 0.01; ***p < 0.001). [file HIPO-36-0-s004.tif]

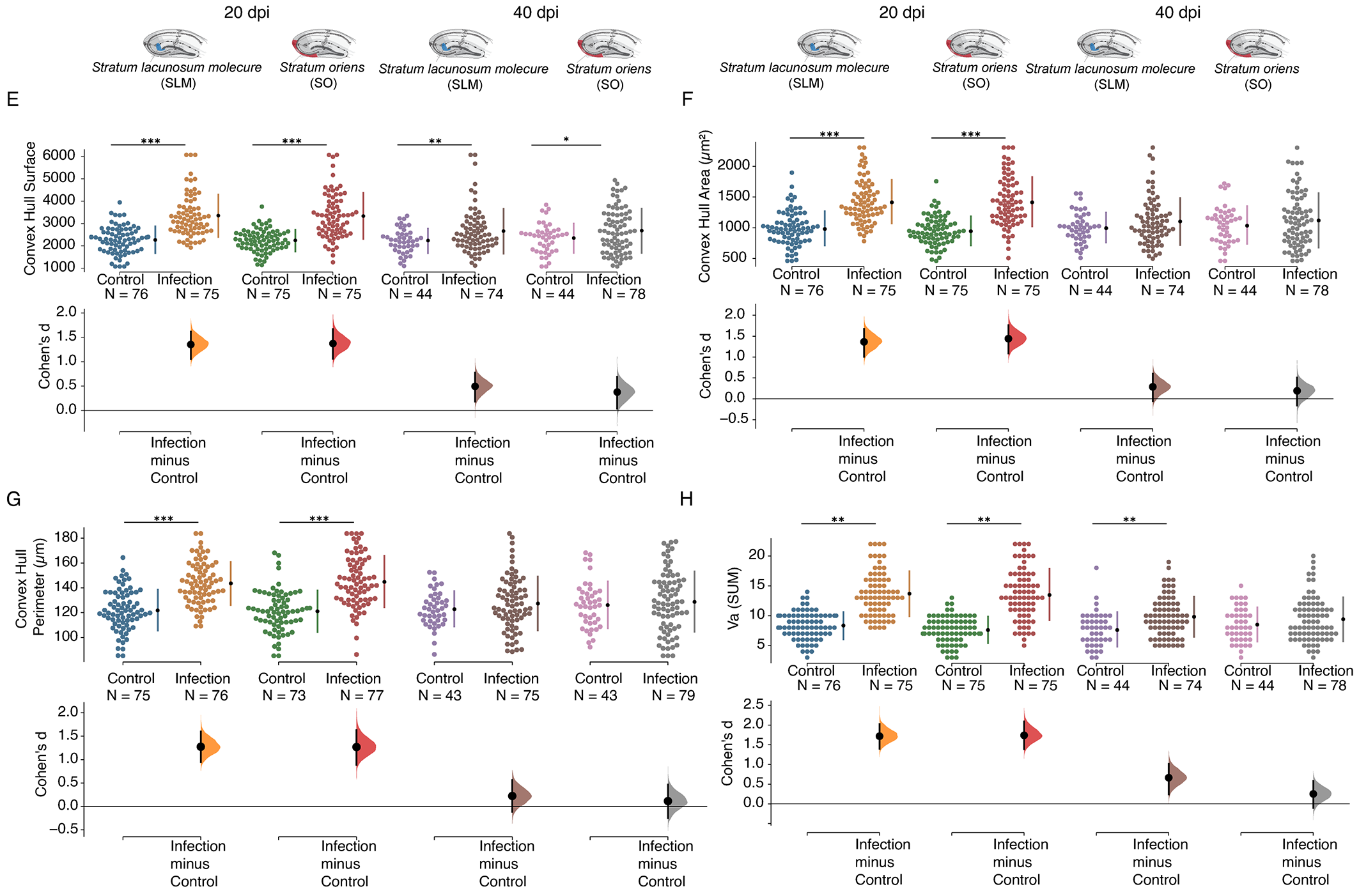

Supplement: Supplementary file 2 — Figure S2: Three‐dimensional reconstruction of astrocytes from Swiss albino mice infected with Piry virus in the Stratum lacunosum‐moleculare (SLM) and Stratum oriens (SO) layers, in the time windows of 20‐ and 40‐days post‐infection (dpi). The graphs represent morphological measures extracted from the 3D reconstructions: (E) Convex Hull Surface (μm2), (F) Convex Hull Area (μm2), (G) Convex Hull Perimeter (μm2), (H) Vertex A (Va (SUM)). The lower graphs show the effect sizes (Cohen's d), highlighting the magnitude of the differences between the groups (Infection—Control). The analyses demonstrated regional and temporal morphological changes in astrocytes in response to viral infection. N indicates the number of cells analyzed per group. Asterisks indicate statistically significant differences (*p < 0.05; **p < 0.01; ***p < 0.001). [file HIPO-36-0-s014.tif]

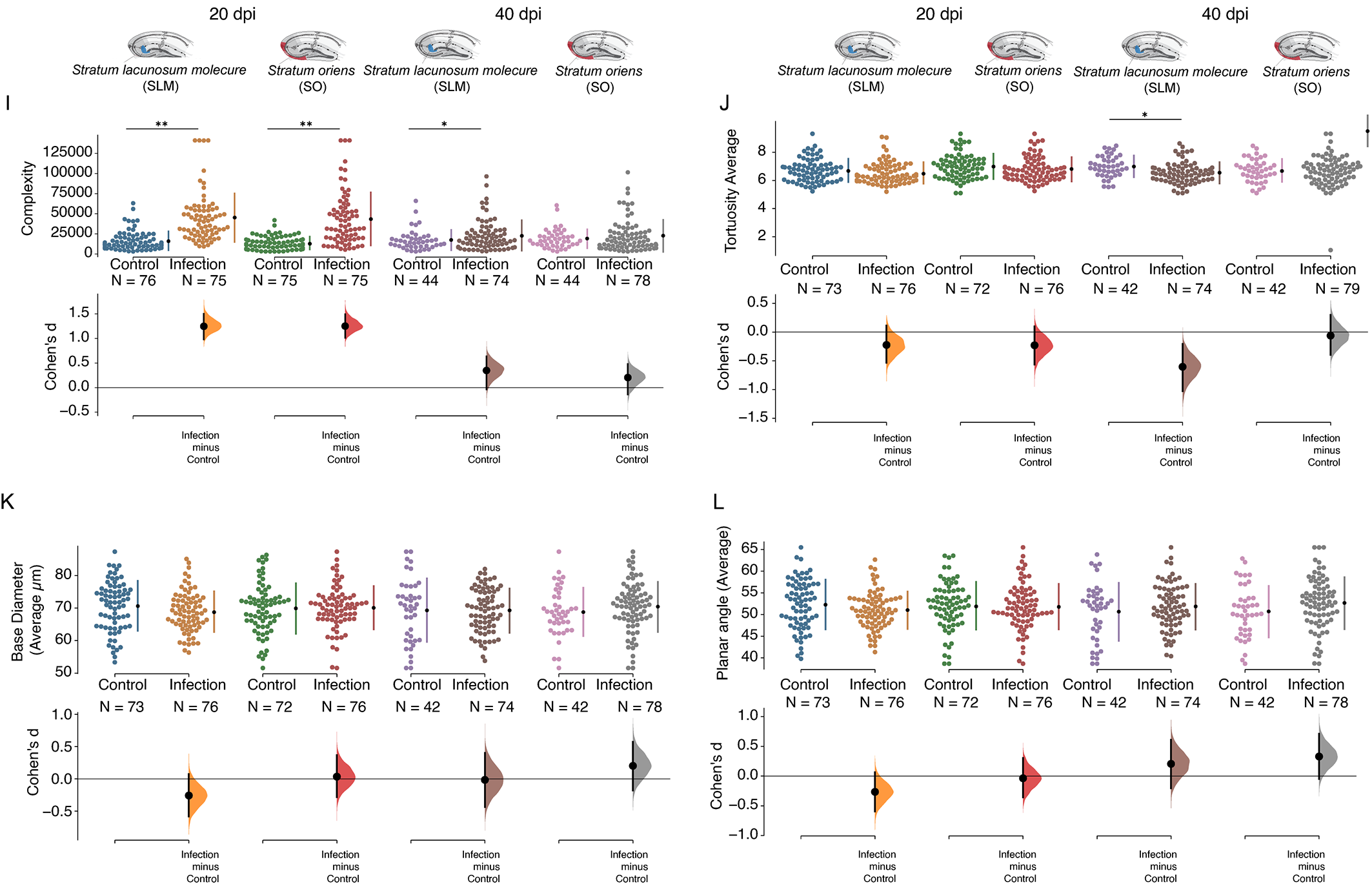

Supplement: Supplementary file 3 — Figure S3: Three‐dimensional reconstruction of astrocytes from Swiss albino mice infected with Piry virus in the Stratum lacunosum‐moleculare (SLM) and Stratum oriens (SO) layers, in the temporal windows of 20‐ and 40‐days post‐infection (dpi). The graphs represent morphological measures extracted from the 3D reconstructions: morphological complexity (I), number of trees (J), base diameter (K) and mean tortuosity (L). The lower graphs show the effect sizes (Cohen's d), highlighting the magnitude of the differences between the groups (Infection—Control). The analyses demonstrated regional and temporal morphological changes in astrocytes in response to viral infection. N indicates the number of cells analyzed per group. Asterisks indicate statistically significant differences (*p < 0.05; **p < 0.01; ***p < 0.001). [file HIPO-36-0-s016.tif]

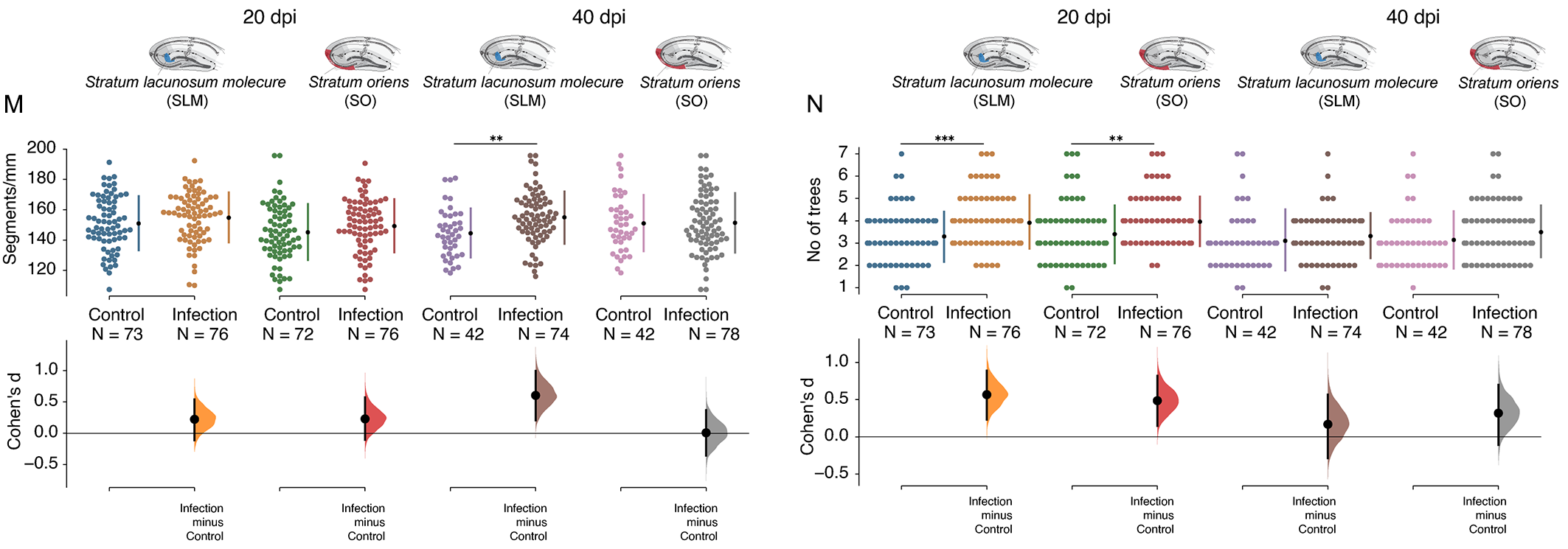

Supplement: Supplementary file 4 — Figure S4: Three‐dimensional reconstruction of astrocytes from Swiss albino mice infected with Piry virus in the Stratum lacunosum‐moleculare (SLM) and Stratum oriens (SO) layers, at 20‐ and 40‐days post‐infection (dpi). The graphs represent morphological measures extracted from the 3D reconstructions: (M) segments/mm and (N) planar angle (average). The lower graphs show the effect sizes (Cohen's d), highlighting the magnitude of the differences between the groups (Infection—Control). The analyses demonstrated regional and temporal morphological changes in astrocytes in response to viral infection. N indicates the number of cells analyzed per group. Asterisks indicate statistically significant differences (*p < 0.05; **p < 0.01; ***p < 0.001). [file HIPO-36-0-s006.tif]
