# Supplementary material for: Layer‐Specific Astrocyte Morphological Responses in the CA3 Hippocampus Region During Piry Virus‐Induced Encephalitis
Source: Hippocampus. 2026 Feb 22;36(2):e70085. doi: 10.1002/hipo.70085 (PMC12926523; doi:10.1002/hipo.70085)
Supplement: Supplementary file 5 — Table S1: Morphometric parameters of three‐dimensional reconstructed astrocytes. [file HIPO-36-0-s007.docx]

# Table S1. Morphometric Parameters of Three-Dimensional Reconstructed Astrocytes

| Morphometric Parameter | Definition |
| --- | --- |
| Segment | Any portion of microglial branched structure with endings that are either nodes or terminations with no intermediate nodes. |
| Segments/mm | Number of segments/total length of the segments expressed in millimeters. |
| Number of Trees | Number of trees in the astrocyte. |
| Total Number of Segments | Refers to the total number of segments in the tree. |
| Branch Length | Total length of the line segments used to trace the branch of interest. |
| Total Branch Length | Total length for all branches in the tree. Mean = [Length] / [Number of branches]. |
| Tortuosity | [Actual length of the segment]/[Distance between the endpoints of the segment]. The smallest value is 1; this represents a straight segment. |
| Surface Area | Computed by modeling each branch as a frustum (truncated right circular cone). |
| Branch Volume | Computed by modeling each piece of each branch as a frustum. |
| Total Branch Volume | Total volume for all branches in the tree. |
| Base Diameter of Primary Branch | Diameter at the start of the 1st segment. |
| Planar Angle | Computed based on the endpoints of the segments. Refers to the change in direction of a segment relative to the previous segment. |
| Fractal Dimension | The 'k-dim' of the fractal analysis, describes how the structure of interest fills space. Differences in K-dim suggest morphological dissimilarities. |
| Complexity | Complexity = [Sum of the terminal orders + Number of terminals] × [Total branch length / Number of primary branches]. |
| Convex Hull | Measures the size of the branching field by interpreting the structure as a solid object, defined by its convex-hull volume, surface area, area, and perimeter. |
