# Supplementary material for: Layer‐Specific Astrocyte Morphological Responses in the CA3 Hippocampus Region During Piry Virus‐Induced Encephalitis
Source: Hippocampus. 2026 Feb 22;36(2):e70085. doi: 10.1002/hipo.70085 (PMC12926523; doi:10.1002/hipo.70085)
Supplement: Supplementary file 6 — Table S2: Summary of multivariate statistical procedures. [file HIPO-36-0-s017.docx]

| **Step** | **Objective / Description** | **Method / Software** | **References** |
| --- | --- | --- | --- |
| 1. Identification of Multivariate Outliers | Detect atypical cases that may influence subsequent analyses. | Mahalanobis Distance (χ², p < 0.001) – IBM SPSS 2019 | Tabachnick & Fidell (2013) |
| 2. Outlier Treatment | Correction of extreme values through Winsorization (1st and 99th percentiles). | Winsorization based on MAH – IBM SPSS 2019 | Dixon (1980) |
| 3. Selection of Multimodal Variables | Filtering variables with a Multimodality Index (MMI > 0.55). | Calculation of MMI = [(M3² + 1)/((M4 + 3(n−1)²)/((n−2)n−3))] | Schweitzer & Renehan (1997) |
| 4. Hierarchical Cluster Analysis | Explore the internal data structure and identify grouping patterns. | Euclidean Distance (z-score) + Ward’s Method – R (MultivariateAnalysis package) | Wright (2022) |
| 5. Cluster Validation | Determine the optimal number of clusters and assess consistency. | NbClust Index (k = 2) – R (MultivariateAnalysis package) | Wright (2022) |
| 6. Association between Clusters and Experimental Groups | Assess the relationship between clusters and experimental groups. | Chi-square Test + Cramer’s V – SPSS 2019 | Pimentel-Gomes (2023) |
| 7. Linear Discriminant Analysis (LDA) | Validate and interpret the variables that best discriminate the clusters. | LDA – SPSS 2019 / Bioestat 5.0 | Vicini (2005); Ayres (2005) |
| 8. Factorial Comparison Tests | Examine the effects of fixed factors (Group, Time, Layer) on dependent variables. | Factorial MANOVA (α < 0.05) – JASP 18.1 | Tabachnick & Fidell (2013); Finch & French (2015); Fávero (2017) |

Table S2 – Summary of Multivariate Statistical Procedures
