# Supplementary material for: Layer‐Specific Astrocyte Morphological Responses in the CA3 Hippocampus Region During Piry Virus‐Induced Encephalitis
Source: Hippocampus. 2026 Feb 22;36(2):e70085. doi: 10.1002/hipo.70085 (PMC12926523; doi:10.1002/hipo.70085)
Supplement: Supplementary file 7 — Table S3: Three‐dimensional morphometric analysis of astrocytes from Swiss albino mice infected with the Piry virus, reconstructed in the stratum lacunosum moleculare (SLM) and stratum oriens (SO) regions at two post‐infection time points: 20 and 40 days (dpi). Data are presented as mean ± standard deviation, and comparisons between control and infected groups were performed using Welch's corrected Student's t‐test for unequal variances. The table also provides t‐values, degrees of freedom (df), p‐values, and effect sizes (Cohen's d). p‐values < 0.05 were considered statistically significant. [file HIPO-36-0-s001.docx]

| **Tabela S3 -** Three-dimensional morphometric analysis of astrocytes from *Swiss albino* mice infected with the Piry virus, reconstructed in the stratum lacunosum moleculare (SLM) and stratum oriens (SO) regions at two post-infection time points: 20 and 40 days (dpi). Data are presented as mean ± standard deviation, and comparisons between control and infected groups were performed using Welch’s corrected Student’s t-test for unequal variances. The table also provides t-values, degrees of freedom (df), p-values, and effect sizes (Cohen’s d). p-values < 0.05 were considered statistically significant. | | | | | | | | | |
| --- | --- | --- | --- | --- | --- | --- | --- | --- | --- |
| **Variable** | **Layer** | **Window** | **Group** | **Mean** | **Standard Deviation** | **Statistic (t)** | **df** | **p** | **Cohen's d** |
| k-Dim | SLM | 20 dpi | Control | 1.07 | 0.03 | -11.61 | 148.8 | < .001 | -1.90 |
|  |  |  | Infection | 1.13 | 0.03 |  |  |  |  |
|  | SLM | 40 dpi | Control | 1.08 | 0.03 | -2.14 | 107.6 | 0.034 | -0.40 |
|  |  |  | Infection | 1.09 | 0.04 |  |  |  |  |
|  | SO | 20 dpi | Control | 1.07 | 0.03 | -8.81 | 127.1 | < .001 | -1.46 |
|  |  |  | Infection | 1.12 | 0.04 |  |  |  |  |
|  | SO | 40 dpi | Control | 1.08 | 0.03 | -0.65 | 106.7 | 0.516 | -0.12 |
|  |  |  | Infection | 1.08 | 0.04 |  |  |  |  |
| Length Average (μm) | SLM | 20 dpi | Control | 1.56 | 0.05 | -4.21 | 144.5 | < .001 | -0.68 |
|  |  |  | Infection | 1.59 | 0.04 |  |  |  |  |
|  | SLM | 40 dpi | Control | 1.57 | 0.04 | 5.41 | 59.5 | 0.628 | 0.09 |
|  |  |  | Infection | 1.57 | 0.05 |  |  |  |  |
|  | SO | 20 dpi | Control | 1.56 | 0.06 | -13.95 | 142 | 0.184 | -0.22 |
|  |  |  | Infection | 1.57 | 0.05 |  |  |  |  |
|  | SO | 40 dpi | Control | 1.57 | 0.05 | -0.04 | 93.8 | 0.547 | -0.11 |
|  |  |  | Infection | 1.57 | 0.05 |  |  |  |  |
| VB (SUM) [2] | SLM | 20 dpi | Control | 7.86 | 3.54 | -7.99 | 139 | < .001 | -1.30 |
|  |  |  | Infection | 13.19 | 4.59 |  |  |  |  |
|  | SLM | 40 dpi | Control | 7.11 | 2.86 | -2.52 | 115.3 | 0.011 | -0.46 |
|  |  |  | Infection | 8.81 | 4.30 |  |  |  |  |
|  | SO | 20 dpi | Control | 7.51 | 3.87 | -6.83 | 132.7 | < .001 | -1.12 |
|  |  |  | Infection | 12.83 | 5.52 |  |  |  |  |
|  | SO | 40 dpi | Control | 7.93 | 2.70 | - | 119.8 | 0.117 | -0.27 |
|  |  |  | Infection | 9.01 | 4.86 |  |  |  |  |
| VC (SUM) | SLM | 20 dpi | Control | 3.41 | 1.83 | -7.35 | 127 | < .001 | -1.20 |
|  |  |  | Infection | 6.25 | 2.81 |  |  |  |  |
|  | SLM | 40 dpi | Control | 2.96 | 1.97 | -3.50 | 106.2 | < .001 | -0.65 |
|  |  |  | Infection | 4.37 | 2.37 |  |  |  |  |
|  | SO | 20 dpi | Control | 2.88 | 1.62 | -8.78 | 109.2 | < .001 | -1.43 |
|  |  |  | Infection | 6.53 | 3.22 |  |  |  |  |
|  | SO | 40 dpi | Control | 3.38 | 2.27 | -1.41 | 106.1 | 0.162 | -0.26 |
|  |  |  | Infection | 4.03 | 2.73 |  |  |  |  |
| TREE Volume (Average μm³) | SLM | 20 dpi | Control | 10.4 | 2.00 | -3.17 | 148.4 | 0.002 | -0.52 |
|  |  |  | Infection | 11.39 | 1.85 |  |  |  |  |
|  | SLM | 40 dpi | Control | 11.55 | 2.10 | 4.70 | 71.6 | < .001 | 0.92 |
|  |  |  | Infection | 9.87 | 1.50 |  |  |  |  |
|  | SO | 20 dpi | Control | 11.02 | 2.10 | -2.24 | 148 | 0.001 | -0.53 |
|  |  |  | Infection | 12.14 | 2.12 |  |  |  |  |
|  | SO | 40 dpi | Control | 11.48 | 2.19 | 1.95 | 72.7 | 0.055 | 0.38 |
|  |  |  | Infection | 10.75 | 1.63 |  |  |  |  |
| Surface Area (Average μm²) | SLM | 20 dpi | Control | 1.24 | 0.06 | 2.62 | 134.8 | 0.01 | 0.43 |
|  |  |  | Infection | 1.22 | 0.04 |  |  |  |  |
|  | SLM | 40 dpi | Control | 1.21 | 0.06 | -1.21 | 77.1 | 0.23 | -0.24 |
|  |  |  | Infection | 1.23 | 0.05 |  |  |  |  |
|  | SO | 20 dpi | Control | 1.22 | 0.06 | 1.20 | 140 | 0.222 | -0.20 |
|  |  |  | Infection | 1.21 | 0.05 |  |  |  |  |
|  | SO | 40 dpi | Control | 1.22 | 0.06 | -0.40 | 82.3 | 0.693 | -0.08 |
|  |  |  | Infection | 1.23 | 0.05 |  |  |  |  |
