# Supplementary material for: Layer‐Specific Astrocyte Morphological Responses in the CA3 Hippocampus Region During Piry Virus‐Induced Encephalitis
Source: Hippocampus. 2026 Feb 22;36(2):e70085. doi: 10.1002/hipo.70085 (PMC12926523; doi:10.1002/hipo.70085)
Supplement: Supplementary file 9 — Table S5: Discriminant analysis results for the control 20 dpi SO group. [file HIPO-36-0-s009.docx]

# Table S5. Discriminant Analysis Results for the Control 20 dpi SO Group

Includes descriptive statistics, significance tests, and classification functions.

| Sampling |
| --- |
| Total number of valid cases: 75 |
| Correct classification rate (%): 97.3 |
| Discriminant Functions |
| Eigenvalues (explained variance) |
| Function 1: 2.080 (61.54%) |
| Function 2: 1.300 (38.46%) |
| Canonical Correlation |
| Function 1: 0.822 |
| Function 2: 0.752 |
| Significance Tests |
| Equality of Means (Wilks' Lambda) |
| Zscore(Complexity): Λ = 0.429, F(2,72) = 47.96, p < 0.001 |
| Zscore(Convex Hull Volume): Λ = 0.336, F(2,72) = 71.18, p < 0.001 |
| Wilks' Lambda for Functions |
| Functions 1 and 2: Λ = 0.141, χ²(4) = 140.00, p < 0.001 |
| Function 2: Λ = 0.435, χ²(1) = 59.56, p < 0.001 |
| Classification Function Coefficients (Fisher) |
| Group 1 |
| Zscore(Complexity): -0.789 |
| Zscore(Convex Hull Volume): -2.502 |
| Constant: -2.445 |
| Group 2 |
| Zscore(Complexity): -0.831 |
| Zscore(Convex Hull Volume): 2.631 |
| Constant: -2.303 |
| Group 3 |
| Zscore(Complexity): 3.873 |
| Zscore(Convex Hull Volume): -1.490 |
| Constant: -4.410 |

Note: Λ = Wilks' Lambda. All tests were two-tailed. The classification rate refers to the accuracy of the discriminant model. p-values < 0.001 indicate statistical significance at the 99.9% confidence level.
