# Supplementary material for: Layer‐Specific Astrocyte Morphological Responses in the CA3 Hippocampus Region During Piry Virus‐Induced Encephalitis
Source: Hippocampus. 2026 Feb 22;36(2):e70085. doi: 10.1002/hipo.70085 (PMC12926523; doi:10.1002/hipo.70085)
Supplement: Supplementary file 10 — Table S6: Discriminant analysis results for the control 40 dpi SLM group. [file HIPO-36-0-s005.docx]

# Table S6. Discriminant Analysis Results for the Control 40 dpi SLM Group

Includes descriptive statistics, significance tests, and classification functions.

| Sampling |
| --- |
| Total number of valid cases: 45 |
| Correct classification rate (%): 91.1 |
| Discriminant Functions |
| Eigenvalues (explained variance) |
| Function 1: 4.116 (80.95%) |
| Function 2: 0.969 (19.05%) |
| Canonical Correlation |
| Function 1: 0.897 |
| Function 2: 0.701 |
| Significance Tests |
| Equality of Means (Wilks' Lambda) |
| Zscore(Complexity): Λ = 0.378, F(3,41) = 22.49, p < 0.001 |
| Zscore(Convex Hull Volume): Λ = 0.236, F(3,41) = 44.29, p < 0.001 |
| Wilks' Lambda for Functions |
| Functions 1 and 2: Λ = 0.099, χ²(6) = 94.70, p < 0.001 |
| Function 2: Λ = 0.508, χ²(2) = 27.77, p < 0.001 |
| Classification Function Coefficients (Fisher) |
| Group 1 |
| Zscore(Complexity): -2.287 |
| Zscore(Convex Hull Volume): -4.741 |
| Constant: -5.038 |
| Group 2 |
| Zscore(Complexity): -0.024 |
| Zscore(Convex Hull Volume): -1.284 |
| Constant: -1.594 |
| Group 3 |
| Zscore(Complexity): 3.946 |
| Zscore(Convex Hull Volume): 4.687 |
| Constant: -6.892 |
| Group 4 |
| Zscore(Complexity): -1.496 |
| Zscore(Convex Hull Volume): 4.589 |
| Constant: -4.665 |

Note: Λ = Wilks' Lambda. All tests were two-tailed. The classification rate refers to the model's accuracy. p-values < 0.001 indicate statistical significance at the 99.9% confidence level.
