# Supplementary material for: Layer‐Specific Astrocyte Morphological Responses in the CA3 Hippocampus Region During Piry Virus‐Induced Encephalitis
Source: Hippocampus. 2026 Feb 22;36(2):e70085. doi: 10.1002/hipo.70085 (PMC12926523; doi:10.1002/hipo.70085)
Supplement: Supplementary file 11 — Table S7: Discriminant analysis results for the control 40 dpi SO group. [file HIPO-36-0-s012.docx]

# Table S7. Discriminant Analysis Results for the Control 40 dpi SO Group

Includes descriptive statistics, significance tests, and classification functions.

| Sampling |
| --- |
| Total number of valid cases: 45 |
| Correct classification rate (%): 97.8 |
| Discriminant Functions |
| Eigenvalues (explained variance) |
| Function 1: 1.990 (69.85%) |
| Function 2: 0.859 (30.15%) |
| Canonical Correlation |
| Function 1: 0.816 |
| Function 2: 0.680 |
| Significance Tests |
| Equality of Means (Wilks' Lambda) |
| Zscore(Complexity): Λ = 0.469, F(2,42) = 23.73, p < 0.001 |
| Zscore(Convex Hull Volume): Λ = 0.364, F(2,42) = 36.67, p < 0.001 |
| Wilks' Lambda for Functions |
| Functions 1 and 2: Λ = 0.180, χ²(4) = 71.19, p < 0.001 |
| Function 2: Λ = 0.538, χ²(1) = 25.74, p < 0.001 |
| Classification Function Coefficients (Fisher) |
| Group 1 |
| Zscore(Complexity): -1.224 |
| Zscore(Convex Hull Volume): 2.982 |
| Constant: -3.119 |
| Group 2 |
| Zscore(Complexity): 1.546 |
| Zscore(Convex Hull Volume): 0.586 |
| Constant: -1.763 |
| Group 3 |
| Zscore(Complexity): -1.429 |
| Zscore(Convex Hull Volume): -2.610 |
| Constant: -2.946 |

Note: Λ = Wilks' Lambda. All tests were two-tailed. The classification rate refers to the model's accuracy. p-values < 0.001 indicate statistical significance at the 99.9% confidence level.
