# Supplementary material for: Layer‐Specific Astrocyte Morphological Responses in the CA3 Hippocampus Region During Piry Virus‐Induced Encephalitis
Source: Hippocampus. 2026 Feb 22;36(2):e70085. doi: 10.1002/hipo.70085 (PMC12926523; doi:10.1002/hipo.70085)
Supplement: Supplementary file 12 — Table S8: Discriminant analysis results for the post‐infection 20 dpi SLM group. [file HIPO-36-0-s010.docx]

# Table S8. Discriminant Analysis Results for the Post-Infection 20 dpi SLM Group

Includes descriptive statistics, significance tests, and classification functions.

| Sampling |
| --- |
| Total number of valid cases: 75 |
| Correct classification rate (%): 97.3 |
| Discriminant Functions |
| Eigenvalues (explained variance) |
| Function 1: 7.357 (99.27%) |
| Function 2: 0.054 (0.73%) |
| Canonical Correlation |
| Function 1: 0.938 |
| Function 2: 0.227 |
| Significance Tests |
| Equality of Means (Wilks' Lambda) |
| Zscore(Complexity): Λ = 0.269, F(2,72) = 98.05, p < 0.001 |
| Zscore(Convex Hull Volume): Λ = 0.380, F(2,72) = 58.81, p < 0.001 |
| Wilks' Lambda for Functions |
| Functions 1 and 2: Λ = 0.113, χ²(4) = 155.59, p < 0.001 |
| Function 2: Λ = 0.948, χ²(1) = 3.79, p = 0.051 |
| Classification Function Coefficients (Fisher) |
| Group 1 |
| Zscore(Complexity): 11.918 |
| Zscore(Convex Hull Volume): 9.485 |
| Constant: -22.524 |
| Group 2 |
| Zscore(Complexity): 1.103 |
| Zscore(Convex Hull Volume): 0.318 |
| Constant: -1.237 |
| Group 3 |
| Zscore(Complexity): -3.763 |
| Zscore(Convex Hull Volume): -2.557 |
| Constant: -2.989 |

Note: Λ = Wilks' Lambda. All tests were two-tailed. Function 2 was not statistically significant (p = 0.051). p-values < 0.001 indicate statistical significance at the 99.9% confidence level.
