# Supplementary material for: Layer‐Specific Astrocyte Morphological Responses in the CA3 Hippocampus Region During Piry Virus‐Induced Encephalitis
Source: Hippocampus. 2026 Feb 22;36(2):e70085. doi: 10.1002/hipo.70085 (PMC12926523; doi:10.1002/hipo.70085)
Supplement: Supplementary file 13 — Table S9: Discriminant analysis results for the post‐infection 20 dpi SO group. [file HIPO-36-0-s002.docx]

# Table S9. Discriminant Analysis Results for the Post-Infection 20 dpi SO Group

Includes descriptive statistics, significance tests, and classification functions.

| Sampling |
| --- |
| Total number of valid cases: 76 |
| Correct classification rate (%): 98.7 |
| Discriminant Functions |
| Eigenvalues (explained variance) |
| Function 1: 2.925 (63.29%) |
| Function 2: 1.696 (36.71%) |
| Canonical Correlation |
| Function 1: 0.863 |
| Function 2: 0.793 |
| Significance Tests |
| Equality of Means (Wilks' Lambda) |
| Zscore(Complexity): Λ = 0.286, F(3,72) = 59.96, p < 0.001 |
| Zscore(Convex Hull Volume): Λ = 0.371, F(3,72) = 40.76, p < 0.001 |
| Wilks' Lambda for Functions |
| Functions 1 and 2: Λ = 0.094, χ²(6) = 169.87, p < 0.001 |
| Function 2: Λ = 0.371, χ²(2) = 71.42, p < 0.001 |
| Classification Function Coefficients (Fisher) |
| Group 1 |
| Zscore(Complexity): -2.111 |
| Zscore(Convex Hull Volume): -0.475 |
| Constant: -2.282 |
| Group 2 |
| Zscore(Complexity): -2.880 |
| Zscore(Convex Hull Volume): 5.819 |
| Constant: -6.003 |
| Group 3 |
| Zscore(Complexity): 2.169 |
| Zscore(Convex Hull Volume): -1.127 |
| Constant: -1.872 |
| Group 4 |
| Zscore(Complexity): 10.475 |
| Zscore(Convex Hull Volume): -4.654 |
| Constant: -12.725 |

Note: Λ = Wilks' Lambda. All tests were two-tailed. The classification rate refers to the model's accuracy. p-values < 0.001 indicate statistical significance at the 99.9% confidence level.
