# Supplementary material for: Layer‐Specific Astrocyte Morphological Responses in the CA3 Hippocampus Region During Piry Virus‐Induced Encephalitis
Source: Hippocampus. 2026 Feb 22;36(2):e70085. doi: 10.1002/hipo.70085 (PMC12926523; doi:10.1002/hipo.70085)
Supplement: Supplementary file 14 — Table S10: Discriminant analysis results for the post‐infection 40 dpi SLM group. [file HIPO-36-0-s013.docx]

# Table S10. Discriminant Analysis Results for the Post-Infection 40 dpi SLM Group

Includes descriptive statistics, significance tests, and classification functions.

| Sampling |
| --- |
| Total number of valid cases: 73 |
| Correct classification rate (%): 98.6 |
| Discriminant Functions |
| Eigenvalues (explained variance) |
| Function 1: 8.272 (62.20%) |
| Function 2: 5.026 (37.80%) |
| Canonical Correlation |
| Function 1: 0.945 |
| Function 2: 0.913 |
| Significance Tests |
| Equality of Means (Wilks' Lambda) |
| Zscore(Complexity): Λ = 0.154, F(3,69) = 126.55, p < 0.001 |
| Zscore(Convex Hull Volume): Λ = 0.124, F(3,69) = 162.60, p < 0.001 |
| Wilks' Lambda for Functions |
| Functions 1 and 2: Λ = 0.018, χ²(6) = 277.59, p < 0.001 |
| Function 2: Λ = 0.166, χ²(2) = 123.93, p < 0.001 |
| Classification Function Coefficients (Fisher) |
| Group 1 |
| Zscore(Complexity): -3.568 |
| Zscore(Convex Hull Volume): -1.547 |
| Constant: -2.767 |
| Group 2 |
| Zscore(Complexity): 2.548 |
| Zscore(Convex Hull Volume): -1.370 |
| Constant: -1.899 |
| Group 3 |
| Zscore(Complexity): 16.533 |
| Zscore(Convex Hull Volume): -5.235 |
| Constant: -21.872 |
| Group 4 |
| Zscore(Complexity): -8.801 |
| Zscore(Convex Hull Volume): 32.125 |
| Constant: -63.814 |

Note: Λ = Wilks' Lambda. All tests were two-tailed. The classification rate refers to the model's accuracy. p-values < 0.001 indicate statistical significance at the 99.9% confidence level.
