# Supplementary material for: Layer‐Specific Astrocyte Morphological Responses in the CA3 Hippocampus Region During Piry Virus‐Induced Encephalitis
Source: Hippocampus. 2026 Feb 22;36(2):e70085. doi: 10.1002/hipo.70085 (PMC12926523; doi:10.1002/hipo.70085)
Supplement: Supplementary file 15 — Table S11: Discriminant analysis results for the post‐infection 40 dpi SO group. [file HIPO-36-0-s008.docx]

# Table S11. Discriminant Analysis Results for the Post-Infection 40 dpi SO Group

Includes descriptive statistics, significance tests, and classification functions.

| Sampling |
| --- |
| Total number of valid cases: 77 |
| Correct classification rate (%): 94.8 |
| Discriminant Functions |
| Eigenvalues (explained variance) |
| Function 1: 6.345 (99.32%) |
| Function 2: 0.043 (0.68%) |
| Canonical Correlation |
| Function 1: 0.929 |
| Function 2: 0.204 |
| Significance Tests |
| Equality of Means (Wilks' Lambda) |
| Zscore(Complexity): Λ = 0.213, F(2,74) = 136.31, p < 0.001 |
| Zscore(Convex Hull Volume): Λ = 0.544, F(2,74) = 31.03, p < 0.001 |
| Wilks' Lambda for Functions |
| Functions 1 and 2: Λ = 0.130, χ²(4) = 149.68, p < 0.001 |
| Function 2: Λ = 0.959, χ²(1) = 3.11, p = 0.078 |
| Classification Function Coefficients (Fisher) |
| Group 1 |
| Zscore(Complexity): -0.117 |
| Zscore(Convex Hull Volume): 0.261 |
| Constant: -1.123 |
| Group 2 |
| Zscore(Complexity): 11.957 |
| Zscore(Convex Hull Volume): 4.928 |
| Constant: -16.598 |
| Group 3 |
| Zscore(Complexity): -4.244 |
| Zscore(Convex Hull Volume): -2.120 |
| Constant: -3.266 |

Note: Λ = Wilks' Lambda. All tests were two-tailed. The classification rate refers to the model's accuracy. Function 2 was not statistically significant (p = 0.078). p-values < 0.001 indicate statistical significance at the 99.9% confidence level.
