# Supplementary material for: Layer‐Specific Astrocyte Morphological Responses in the CA3 Hippocampus Region During Piry Virus‐Induced Encephalitis
Source: Hippocampus. 2026 Feb 22;36(2):e70085. doi: 10.1002/hipo.70085 (PMC12926523; doi:10.1002/hipo.70085)
Supplement: Supplementary file 16 — Table S12: Hierarchical cluster analyses by experimental group. [file HIPO-36-0-s003.docx]

# Table S12. Hierarchical Cluster Analyses by Experimental Group

| Experimental Group | Number of Clusters | Cluster Sizes (cells) | Hopkins Statistic | Cophenetic Correlation Coefficient | Agglomeration Coefficient |
| --- | --- | --- | --- | --- | --- |
| Control 20 dpi SLM | 4 | 38, 12, 20, 6 | 0.924 (Strong) | 0.656 (Moderate) | 0.976 (Very High) |
| Control 20 dpi SO | 3 | 28, 34, 13 | 0.722 (Moderate) | 0.553 (Moderate) | 0.962 (Very High) |
| Control 40 dpi SLM | 4 | 9, 21, 8, 7 | 0.732 (Moderate) | 0.582 (Moderate) | 0.948 (High) |
| Control 40 dpi SO | 3 | 9, 21, 15 | 0.643 (Moderate) | 0.482 (Low) | 0.949 (High) |
| Post-Infection 20 dpi SLM | 3 | 9, 29, 37 | 0.934 (Strong) | 0.803 (High) | 0.984 (Exceptional) |
| Post-Infection 20 dpi SO | 3 | 36, 12, 22 | 0.604 (Moderate) | 0.434 (Low) | 0.980 (Very High) |
| Post-Infection 40 dpi SLM | 4 | 39, 23, 7, 4 | 0.907 (Strong) | 0.519 (Moderate) | 0.983 (Very High) |
| Post-Infection 40 dpi SO | 3 | 36, 11, 30 | 0.676 (Moderate) | 0.725 (High) | 0.979 (Very High) |

Legend:

1. Hopkins Statistic: > 0.75 = Strong clustering tendency; 0.5–0.75 = Moderate; < 0.5 = Low.

2. Cophenetic Correlation Coefficient: > 0.7 = High fit; 0.5–0.7 = Moderate; < 0.5 = Low.

3. Agglomeration Coefficient: > 0.9 = Very high internal consistency; 0.8–0.9 = Good.
