# Supplementary material for: Layer‐Specific Astrocyte Morphological Responses in the CA3 Hippocampus Region During Piry Virus‐Induced Encephalitis
Source: Hippocampus. 2026 Feb 22;36(2):e70085. doi: 10.1002/hipo.70085 (PMC12926523; doi:10.1002/hipo.70085)
Supplement: Supplementary file 17 — Table S13: Summary of multivariate statistical steps. [file HIPO-36-0-s015.docx]

**Supplementary Table S13 – Summary of Multivariate Statistical Steps**

| **Step** | **Purpose / Description** | **Method/Software** | **References** | |
| --- | --- | --- | --- | --- |
| 1. Identification of multivariate outliers | Detect atypical cases that may influence subsequent analyses. | Mahalanobis distance (χ², p < 0.001) – IBM SPSS 2019 | Tabachnick & Fidell (2013) |  |
| 2. Treatment of outliers | Correction of extreme values by Winsorization (1% and 99% percentiles). | MAH based winsorization – IBM SPSS 2019 | Dixon (1980) | |
| 3. Selection of multimodal variables | Filtering of variables with Multimodality Index (MMI > 0.55). | Cálculo do MMI = [(M3² + 1)/((M4 + 3(n−1)²)/((n−2)n−3))] | Schweitzer & Renehan (1997) | |
| 4. Hierarchical Grouping Analysis | Explore the internal structure of the data and identify clustering patterns. | Euclidean distance (z-score) + Ward-R method (MultivariateAnalisis package) | Wright (2022) | |
| 5. Cluster Validation | Determine optimal number of groupings and check consistency. | Nojena index indices with k = 2; R (MultivariateAnalisis package) | Wright (2022) | |
| 6. Association of Clusters × Experimental Groups | To evaluate the relationship between clusters and experimental groups. | Chi-Square + Cramer's V Test – SPSS 2019 | Pimentel-Gomes (2023) | |
| 7. Linear Discriminant Analysis (LDA) | Validate and interpret the variables that most discriminate the clusters. | LDA – SPSS 2019 / Bioestat 5.0 | Vicini (2005); Ayres (2005) | |
| 8. Factor Comparison Tests | To examine the effects of fixed factors (Group, Time, Layer) on dependent variables. | MANOVA fatorial (α < 0.05) – JASP 18.1 | Tabachnick & Fidell (2013); Finch & French (2015); Fávero (2017) | |
